# Synthesis of novel *N*-cyclopentenyl-lactams using the Aubé reaction

**DOI:** 10.3762/bjoc.11.119

**Published:** 2015-06-23

**Authors:** Madhuri V Shinde, Rohini S Ople, Ekta Sangtani, Rajesh Gonnade, D Srinivasa Reddy

**Affiliations:** 1 Division of Organic Chemistry, CSIR-National Chemical Laboratory, Dr. Homi Bhabha Road, Pune, 411008, India; 2 Center for Material Characterization, CSIR-National Chemical Laboratory, Dr. Homi Bhabha Road, Pune, 411008, India

**Keywords:** Aubé reaction, biological activity, carbocyclic nucleosides, cyclopentenylated lactams, cyclopentylated lactams

## Abstract

A novel and convenient method utilizing the Aubé reaction to access a new class of compounds that are similar to carbocyclic nucleosides is reported. The azido alcohol derived from Vince lactam undergoes the Aubé reaction with various cyclic ketones to give cyclopentenyl-substituted lactams. Upon dihydroxylation, this affords the *N*-cyclopentenyl-lactam compounds in racemic form. Given the numerous uses of nucleosides and related compounds, we were interested in the synthesis of carbocylic nucleoside mimics. The attempts and results are described herein.

## Introduction

**Scheme 1:**
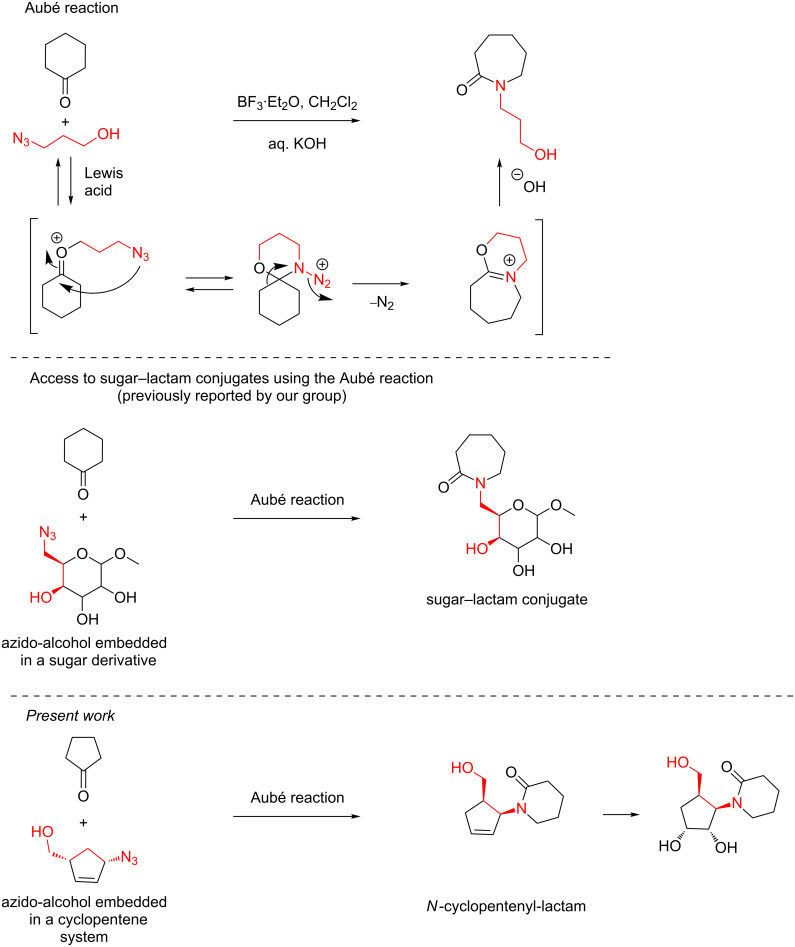
The Aubé reaction and its selected applications.

One popular method for the synthesis of *N*-substituted lactams is the Aubé reaction [1–4]. Over the last few decades, this reaction has gained popularity and resulted in the production of a variety of chemical structures and generated new techniques in chemistry [5–10]. Additionally, it was applied to the synthesis of different natural products of biological importance [11–15]. In the Aubé reaction, an intermolecular reaction takes place between an azido alcohol and a ketone to provide lactams through an in situ*-*generated hemiacetal as a temporary tether. This helps the azide addition in an intramolecular fashion, followed by ring expansion (Scheme 1) [1–4].

**Figure 1:**
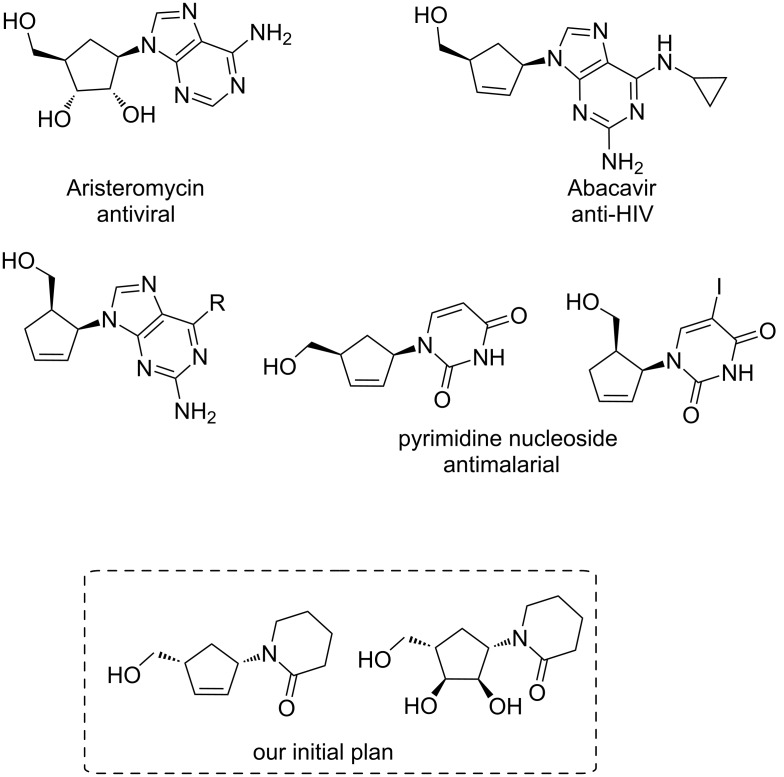
Selective carbocyclic nucleoside analogues from the literature and our initial designs.

Recently, we applied this reaction for the synthesis of sugar–lactam conjugates starting from an azido-alcohol embedded in sugar derivatives and cyclic ketones [16]. In continuation of this work (and also to expand the potential of this chemistry), a new class of compounds were prepared. These cyclopentenoid–lactams, which look like carbocyclic nucleosides, were prepared using the Aubé reaction where it was planned to use an azido alcohol embedded in a cyclopentenoid system. It is well established in the literature that carbocyclic nucleosides and related compounds are important to pharmaceuticals and these compounds have been the focus of many studies and a number of reported syntheses [17–30]. Some selected compounds and their important associated activities are highlighted in Figure 1.

## Results and Discussion

**Scheme 2:**
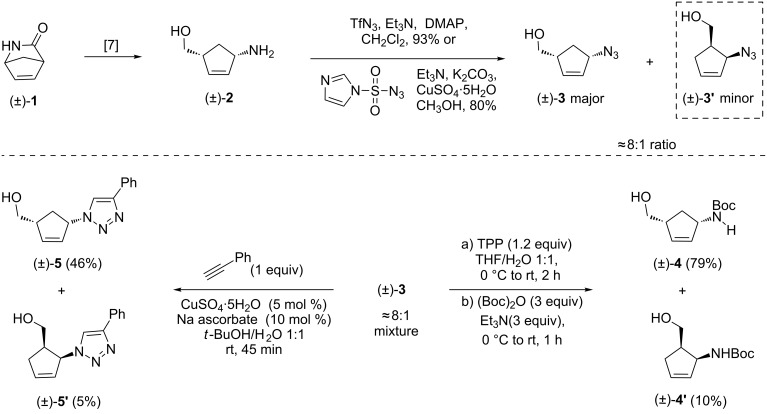
Top: synthesis of azido alcohol derivative **3** and bottom: structural elucidation of the minor diastereomer.

To our knowledge, there are no reports of the synthesis of cyclopentenoid lactams, likely due to the lack of suitable methods to synthesize these compounds. This gives the opportunity to explore the Aubé reaction, which evolves an entirely new family of compounds by using simple chemistry. In this paper, efforts towards target molecules and the details of new chemistry developed during this process are discussed. To begin with, the azido alcohol **3** required for the present work was envisaged from Vince lactam (±)-**1**. The known amino alcohol [31] (prepared from Vince lactam by following literature procedures) was treated with triflyl azide or imidazole sulfonyl azide [32–33] using standard conditions to furnish the desired azido alcohol (±)-**3** as an 8:1 regioisomeric mixture. However, we could neither purify nor isolate compound (±)-**3** in pure form, which was always accompanied by an undesired isomeric compound. To confirm whether it is a diastereomer or regioisomer, we performed two derivatization reactions. Firstly, reduction of the azido functionality in compound (±)-**3** to the corresponding amine followed by Boc protection resulted in two compounds. Fortunately, these were separable by silica gel column chromatography to give compound (±)-**4** and (±)-**4**’ in good yields. The major compound (±)-**4** was known in the literature and the data were compared with that reported [34]. The structure of the minor diastereomer (±)-**4**’ was assigned based on 2D NMR correlations. Secondly, the azido alcohol (±)-**3** was subjected to a click reaction with phenylacetylene under standard conditions and the resulting two compounds, (±)-**5** and (±)-**5’**, were again separable by column chromatography and subsequently characterized. The NMR analysis and comparison with literature data [35] confirmed the structures of the major (±)-**5** and minor isomer (±)-**5**’ (based on 2D NMR analysis), as shown in Scheme 2. These two experiments proved that the major azido alcohol is the desired 1,3-substituted cyclopentenyl derivative (±)-**3**, with the minor being (±)-**3’**.

**Scheme 3:**
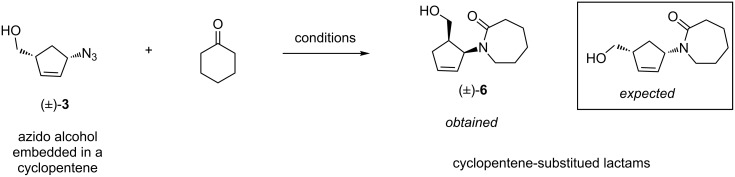
Aubé reaction of cylopentenyl azido alcohol **3** with cyclohexanone.

As we could not separate the isomers of azido alcohol (±)-**3** [36], we decided to proceed with the mixture. The Aubé reaction was performed under similar conditions as the previous work [16]. Cyclohexanone was reacted with azido alcohol (±)-**3** in dichloromethane with varying amounts of BF_3_·OEt_2_ and the results are summarized in Scheme 3 and Table 1. We were able to achieve a moderate yield of cyclic lactam (±)-**6** with excess ketone, Lewis acid and longer reaction times. While this research was underway, Aubé et al. proposed that an unfavorable catalyst–product interaction results in product inhibition, thus deterring the progress of the reaction under catalytic conditions, which leads to the use of excess Lewis acid in super stoichiometric amounts [37–38]. They reported that the use of hexafluoroisopropanol (HFIP) can reduce the Lewis acid requirement. Accordingly, by varying a few conditions, we were able to optimize the reaction as summarized in Table 1. The optimized conditions are: 2.5 equiv of ketone, 4–5 equiv of HFIP and one equiv of BF_3_·OEt_2_ followed by hydrolysis with aq. KOH.

**Table 1:** Optimization of Aubé reaction conditions.

BF_3_·Et_2_O	Solvent	Reaction time	Yield

0.2 equiv	CH_2_Cl_2_	15 h	≈10%
1.5 equiv	CH_2_Cl_2_	1 d	20%
2.5 equiv	CH_2_Cl_2_	1 d	60%
0.2 equiv	HFIP	15 h	≈10%
0.5 equiv	HFIP	7 h	≈10%
1.0 equiv	HFIP	2 h	72%

Upon careful examination of the spectral data and on the basis of the recent results from the Aubé group [37–38], the initially anticipated 1,3-substituted structure was revised to 1,2-substituted cyclopentenoid lactams (±)-**6**, as drawn in Scheme 3.

**Scheme 4:**
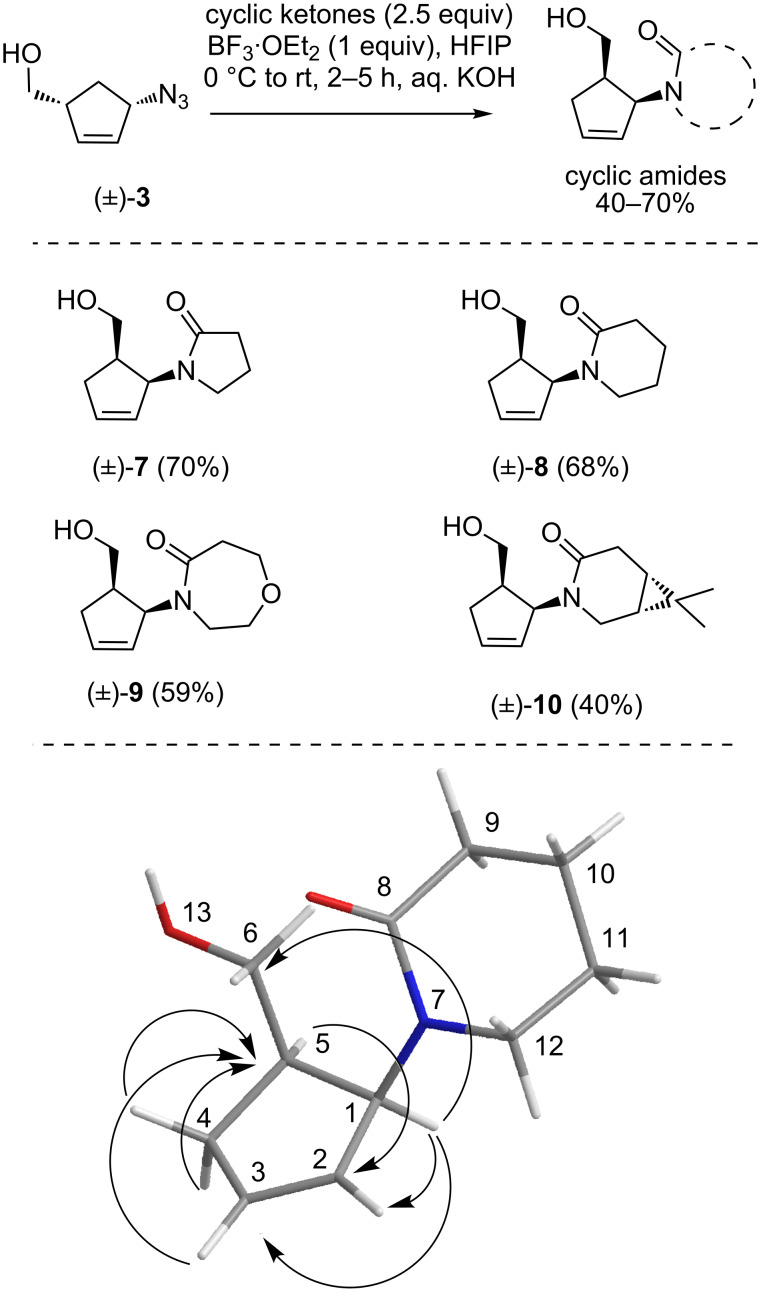
Substrate scope of the reaction: preparation of cyclopentene-substituted lactams and key NMR correlations (HMBC) of compound (±)-**8**.

With the optimized conditions in hand, we then tested the scope of the reaction using different cyclic ketones. Thus, the reaction of the azido alcohol (±)-**3** with cyclobutanone and cyclopentanone afforded the corresponding cyclopentene-substituted lactams (±)-**7** and (±)-**8** in good yields. The 2D NMR analysis and HMBC correlations for one of the products (±)-**8** are shown in Scheme 4 and confirmed the 1,2-substitution in the cyclopentene ring of the product. Similarly, lactam (±)-**9** was prepared from 4-pyranone with (±)-**3** and obtained in moderate yield.

To further broaden the scope of this method, the reaction was also performed with substituted cyclic ketones to give corresponding lactam products in only moderate yields. Unfortunately, in most cases, the desired lactams could not be isolated in pure form and were contaminated with cleaved secondary amides, diastereomers, regioisomers, etc. Finally, the reaction of the constrained symmetrical ketone 6,6-dimethyl-bicyclo[3.1.0]hexan-3-one [39] with (±)-**3** resulted in compound (±)-**10** in 40% yield. Since all synthesized compounds contain a 1,2-substituted cyclopentene ring in their structures, it is suggested that the starting 1,3-azido alcohol **3** undergoes a 1,3-shift to give the corresponding 1,2-azido alcohol under the Lewis acid-mediated reaction conditions.

**Scheme 5:**
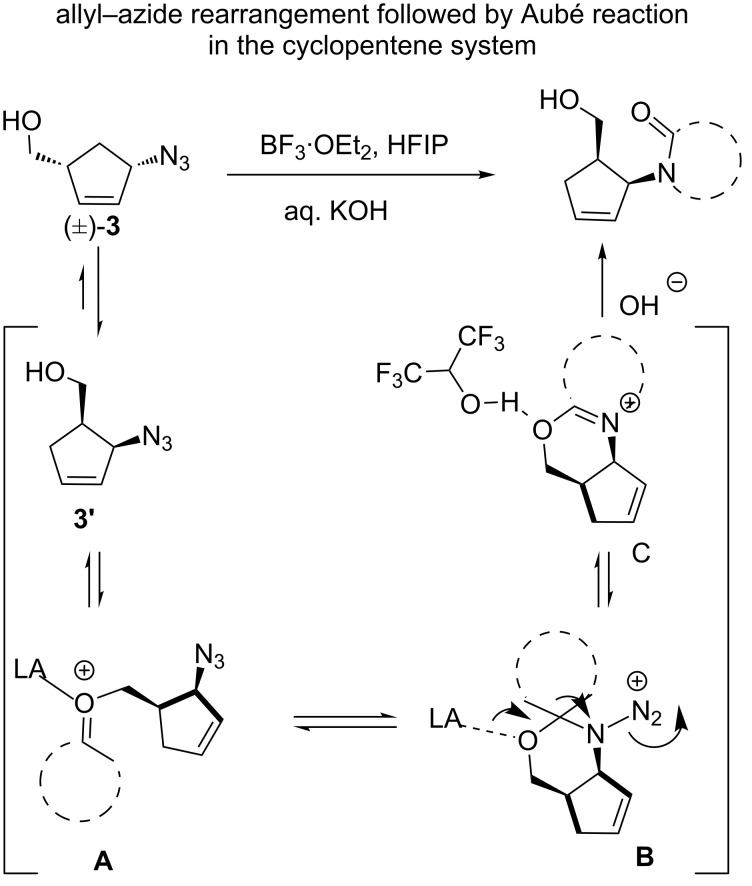
Proposed mechanism for the Aubé reaction for azido alcohols embedded in a cyclopentene system.

We envisage that the 1,2-substituted cyclopentenoid lactams obtained from the Aubé reaction result from the 1,3-allylic rearrangement of compound (±)-**3** to (±)-**3’**. There is a report of allylic azide shift in cyclic sytems by Carell et al. in 2007 [40]. The work of Aubé on AAC (azide–alkyne cycloaddition) chemistry also gives a good indication that equilibrating allylic azide stereoisomers can selectively participate in reactions [37–45]. In 2000, Aubé et al. proposed a mechanism for the intermolecular ring-expansion reaction of hydroxy azides with cyclic ketones. This involves the Lewis acid-promoted formation of an *N-*diazonium intermediate, which undergoes rearrangement to give an iminium ether intermediate that can be hydrolyzed by a base [8,41]. Along these lines we have proposed the mechanism below based on products we isolated from the Aubé reaction (Scheme 5).

**Scheme 6:**
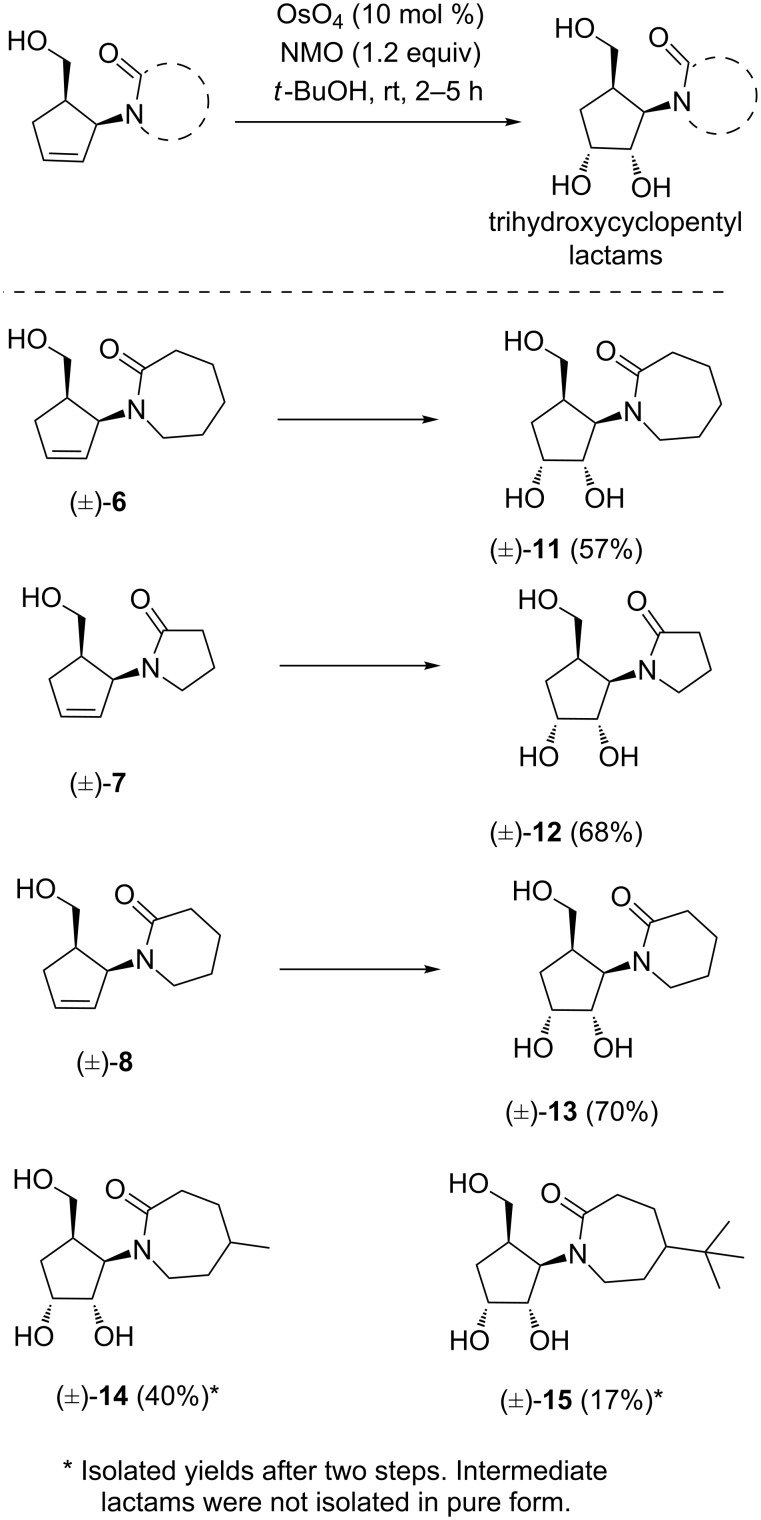
Hydroxylated cyclopentyl-substituted lactams.

After having access to cyclopentenyl-substituted lactams, the double bond present in these products was dihydroxylated under standard conditions using catalytic osmium tetroxide and NMO (Scheme 6) [42–44].

**Figure 2:**
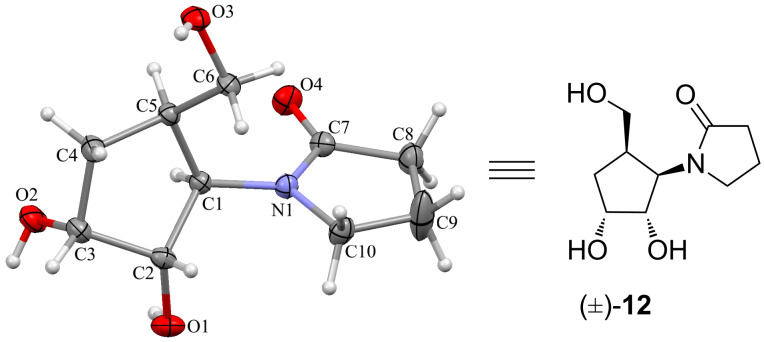
ORTEP plot of triol (±)-**12**.

The corresponding triols (±)-**11** to (±)-**15** were readily formed and obtained as single diastereoisomers, indicating that the reaction proceeded in a highly selective manner. The triols (±)-**14** and (±)-**15** also resulted from corresponding substituted lactams. The observed selectivity of the dihydroxylation can be explained by the reagent approach from the opposite side of both substituents present on the cyclopentene ring. We have assigned the structure based on the spectral data (see Experimental). In addition, crystals of the triol (±)-**12** were obtained and single crystal X-ray analysis further established the assigned structure without any ambiguity, as shown in Figure 2 and Supporting Information File 1 [46–47]. The synthesized trihydroxylated amides (±)-**11**–(±)-**15** can be considered as mimics of 1,2-carbocyclic nucleosides due to close structural resemblance with that of molecules documented in the literature [29–30].

## Conclusion

In conclusion, we have developed an efficient method to synthesize a new class of racemic cyclopentylated lactams using the Aubé reaction where the bases are replaced by lactam rings. We originally planned to synthesize 1,3-substituted cyclopentyl-substituted lactams; however, based on an allyl–azide rearrangement, we obtained lactams with an 1,2-substituted cyclopentane on the nitrogen instead. These compounds can be useful tools as they are structurally close to nucleosides and may have pharmaceutical relevance.

## Experimental

### General procedure for the Aubé reaction using BF_3_·OEt_2_

Boron trifluoride etherate (0.07 mL, 0.71 mmol) was added to a solution of azido alcohol **3** (0.10 g, 0.71 mmol) and ketones (2.0 mmol, 3 equiv) in HFIP (0.2 mL) cooled to 0 °C. The mixture was warmed to room temperature and stirred for 2–5 h. Upon completion, the reaction was quenched with 15% aq. KOH solution diluted with CH_2_Cl_2_ and the mixture was extracted with CH_2_Cl_2_ (3 × 5 mL). The organic layer was washed with brine, dried over anhydrous Na_2_SO_4_ and concentrated in vacuum. The obtained crude oil was subjected to silica gel column chromatography using ethyl acetate/methanol 9:1 as eluent to afford cyclic amides in 40–70% yield.

#### 1-(5-(Hydroxymethyl)cyclopent-2-en-1-yl)azepan-2-one (**6**)

IR ν_max_ (film): 2930, 1618, 1476, 1444, 1039 cm^−1^; ^1^H NMR (500 MHz, CDCl_3_) δ 6.15 (dd, *J* = 3.0, 4.5 Hz, 1H), 5.73 (d, *J* = 1.5 Hz, 1H), 5.34 (d, *J* = 8.0 Hz, 1H), 3.56–3.52 (m, 1H), 3.33 (t, *J* = 10.0 Hz, 1H), 3.21–3.12 (m, 1H), 3.02 (dd, *J* = 6.3, 14.0 Hz, 1H), 2.65–2.56 (series of multiplets, 3H), 2.46–2.39 (m, 1H), 1.93–1.79 (series of multiplets, 4H), 1.72–1.54 (m, 4H); ^13^C NMR (125 MHz, CDCl_3_) δ 178.5, 137.0, 129.6, 62.1, 60.8, 45.3, 45.1, 37.7, 34.4, 29.8, 29.0, 23.4; HRMS calculated for C_12_H_19_NO_2_, [M + Na]^+^: 232.1308, found 232.1316.

#### General procedure for the dihydroxylation reaction

0.2 mL of OsO_4_ (2.5% in *tert*-butanol, 1 mol %) was added to a solution of cyclic lactam (0.27 mmol) and NMO (0.3 mmol, 1.1 equiv) in *tert*-butanol at room temperature and stirred for 2–5 hours. Upon completion of the reaction, Na_2_S (diluted with MeOH) was added, and the mixture was filtered and passed through a short pad of celite. After concentration, the residue was purified by silica gel column chromatography using ethyl acetate/methanol 10:1 as eluent to afford triols in good yield.

#### 1-(2,3-Dihydroxy-5-(hydroxymethyl)cyclopentyl)azepan-2-one (**11**)

IR ν_max_ (film): 3385, 2927, 1623, 1567, 1450, 1358 cm^−1^; ^1^H NMR (400 MHz, CD_3_OD) δ 4.60–4.58 (m, 1H), 4.35 (dd, *J* = 5.0, 10.0 Hz, 1H), 4.08–4.05 (m, 1H), 3.60 (dd, *J* = 7.0, 16.0 Hz, 1H), 3.50–3.36 (series of multiplets, 3H), 2.73–2.67 (m, 1H), 2.56–2.49 (m, 2H), 1.91–1.83 (series of multiplets, 5H), 1.74–1.61 (series of multiplets, 3H); ^13^C NMR (100 MHz, CD_3_OD) δ 178.6, 73.7, 70.5, 62.6, 61.8, 47.3, 37.5, 37.0, 32.9 29.3, 28.0, 23.0; HRMS calculated for C_12_H_21_NO_4_, [M + Na]^+^: 266.1363, found 266.1358.

## Supporting Information

File 1 Experimental procedures, characterization data, and ^1^H and^13^C NMR spectra of relevant compounds.

File 2 Crystal data for (±)-**12**.
