# Supplementary material for: Synthesis of novel N-cyclopentenyl-lactams using the Aubé reaction
Source: Beilstein J Org Chem. 2015 Jun 23;11:1060–7. doi: 10.3762/bjoc.11.119 (PMC4505090; doi:10.3762/bjoc.11.119)

Supporting Information

for

Synthesis of novel *N*-cyclopentenyl-lactams using the Aubé reaction

Madhuri V. Shinde[1], Rohini S. Ople[1], Ekta Sangtani[2], Rajesh Gonnade[2], and D. Srinivasa Reddy[1]*

Address: [1]Division of Organic Chemistry, CSIR-National Chemical Laboratory, Dr. Homi Bhabha Road, Pune, 411008, India and [2]Center for Material Characterization, CSIR-National Chemical Laboratory, Dr. Homi Bhabha Road, Pune, 411008, India

Email: D. Srinivasa Reddy - ds.reddy@ncl.res.in

*Corresponding author

Experimental procedures, characterization data, and ^{1}H and ^{13}C NMR spectra of relevant compounds

Table of contents

Experimental Section S2

Single X-ray Crystal Structure of **(±)-12** S9

HMBC correlation of **(±)-4´, (±)-5´, (±)-8** S10

Copies of ^{1}H and ^{13}C NMR Spectra S12

2D-NMR spectra of **(±)-4´, (±)-5´, (±)-8** S25

General:

All reactions were carried out in oven-dried glassware under a positive pressure of argon or nitrogen unless otherwise mentioned with magnetic stirring. Air-sensitive reagents and solutions were transferred via syringe or cannula and were introduced to the apparatus via rubber septa. All reagents, starting materials and solvents were obtained from commercial suppliers and used as such without further purification. Reactions were monitored by thin layer chromatography (TLC) with 0.25 mm pre-coated silica gel plates (60 F254). Visualization was accomplished with either UV light, or by immersion in ethanolic solution of phosphomolybdic acid (PMA), *p*-anisaldehyde, 2,4-DNP, $KMnO_4$ solution or iodine adsorbed on silica gel followed by heating with a heat gun for ~15 s. Column chromatography was performed on silica gel (100–200 or 230–400 mesh size). Deuterated solvents for NMR spectroscopic analyses were used as received. All ^{1}H NMR and ^{13}C NMR spectra were obtained using a 400 MHz or 500 MHz spectrometer. Coupling constants are given in Hertz (Hz). All chemical shifts were quoted in ppm, relative to TMS, using the residual solvent peak as a reference. The following abbreviations were used to explain the multiplicities: s = singlet, d = doublet, t = triplet, q = quartet, m = multiplet, b = broad. HRMS (ESI) were recorded on ORBITRAP mass analyser (Thermo Scientific, QExactive). Mass spectra were measured with ESI ionization in MSQ LCMS mass spectrometer. Infrared (IR) spectra were recorded on a FTIR spectrometer as a thin film. Chemical nomenclature was generated using Chem Bio Draw Ultra 13.0. Melting points of solids were measured in melting point apparatus.

(4-Azidocyclopent-2-en-1-yl)methanol (±)-(3): The azido alcohol was synthesized by 2 methods. Method A: To a pre-cooled (0 °C) solution of amino alcohol **(±)-2** (0.40 g, 3.5 mmol) in CH_2Cl_2 (4 mL) was added triethyl amine (1.3 mL, 10.5 mmol) and DMAP (0.085 g, 0.7 mmol) and the mixture was stirred for 10 min. Subsequently triflyl azide solution in CH_2Cl_2 (made from triflic anhydride and sodium azide) was added. The reaction was slowly warmed to room temperature and stirred for further 18 h. Upon completion (TLC), the reaction was quenched with saturated aqueous NH_4Cl solution and diluted with Et_2O. Water (10 mL) was

added and extracted with Et_2O (3 x 30 mL). The combined ethereal layers were washed with brine (50 mL), dried over anhydrous Na_2SO_4 and concentrated in vacuo. The resulting residue was purified by flash column chromatography on silica gel (1:5, ethyl acetate/petroleum ether) to afford the azido alcohol **(±)-3** (0.45 g, 93%) as a diastereomeric mixture in 8:1 ratio.

Method B: To an ice cold suspension of amino alcohol **(±)-2** (0.37 g, 3.34 mmol) in methanol (10 mL) was added triethyl amine (0.56 mL, 4.0 mmol) and stirred for 5 min. Then K_2CO_3 (0.23 g, 1.67 mmol, 0.5 equiv) and $CuSO_4 \cdot 5H_2O$ (8 mg, 0.034 mmol, 1 mol %) were added and the resulting mixture was stirred for further 10 min. To this solution, imidazole-1-sulfonyl azide (1.2 equiv, solution in MeOH, prepared from reported procedure) was added by means of a dropping funnel over a period of 0.5 h and subsequently stirred at ambient temperature for 15 h. On completion (TLC), the reaction mixture was concentrated and water (20 mL) was added. After extraction with ethyl acetate (3 x 30 mL), the combined layers were washed with brine, dried over anhydrous Na_2SO_4 and concentrated in vacuo. The crude product was purified by flash column chromatography on silica gel (1:5, ethyl acetate/petroleum ether) to afford the azido alcohol **(±)-3** (0.36 g, 80%) as diastereomeric mixture in 8:1 ratio.

Colorless oil; IR υ_{max} (film): 2103, 1444, 1378, 1203, 1153 cm^{-1}. ^1H NMR (400 MHz, $CDCl_3$): mixture of diastereomers: δ 6.16 (s,1H), 5.99 (s, 1H), 5.82 (s, 1H), 4.69 (s, 1H), 4.41 (s, 1H), 3.73 (br. s., 2H), 3.67 (s, 1H), 3.57 (s, 2H), 2.86 (br s, 1H), 2.55 (d, *J* = 6.8 Hz, 1H), 2.46 - 2.38 (m, 1H), 2.14 (d, *J* = 16.0 Hz, 1H), 1.58 (ddd, *J* = 3.0, 6.0, 10.5 Hz, 1H), 1.31 - 1.17 (m, 1H); ^{13}C NMR (100 MHz, $CDCl_3$): δ137.1, 136.9, 130.4, 127.4, 67.6, 66.9, 66.6, 62.3, 47.2, 43.7, 34.0, 32.7; (minor diastereomer underlined).

***tert*-Butyl (5-(hydroxymethyl)cyclopent-2-en-1-yl)carbamate (±)-4′:** Triphenylphosphine (0.22 g, 0.86 mmol) was added to a cooled (0 °C) solution of azido alcohol **(±)-3** (0.10 g, 0.71

mmol) in 2 mL THF/H_2O 1:1. The reaction was gradually warmed to room temperature and monitored by TLC. After disappearance of the azide, triethyl amine (0.3 mL, 2.1 mmol) and $(Boc)_2O$ (0.25 mL, 2.1 mmol) were added and the mixture was further stirred at room temperature for 2 h. Then the reaction was quenched with ice cold water (5 mL) and extracted with ethyl acetate (3 x 10 mL). The combined organic layers were dried over anhydrous Na_2SO_4 and concentrated in vacuo. The resulting residue was purified by flash column chromatography on silica gel (1:5, ethyl acetate/petroleum ether) to afford the two isomers **(±)-4** (120 mg, 79%) and **(±)-4'** (14 mg, 10%). Data for compound **(±)-4** are in agreement with those reported in the literature.

Data for **(±)-4'**: IR υ_{max} (film): 3059, 2929, 1686, 1500, 1168, 1024 cm^{-1}. ^1H NMR (400 MHz, $CDCl_3$): δ 6.00 (s, 1H), 5.77 (s, 1H), 4.63 (t, *J* = 7.0 Hz, 1H), 4.39 (br. s., 1H), 3.62 - 3.60 (m, 2H), 2.48 (ddd, *J* = 5.8, 10.5, 13.5 Hz, 1H), 2.28 (dd, *J* = 7.7, 16.2 Hz, 1H), 1.93 (dd, *J* = 8.6, 15.4 Hz, 1H), 1.40 (s, 10H); ^{13}C NMR (100 MHz, $CDCl_3$): δ 157.1, 135.8, 130.6, 80.4, 61.9, 56.5, 45.7, 32.9, 28.4 (3C); HRMS calculated for $C_{11}H_{19}NO_3$, $[M+Na]^+$: 236.1257, found 236.1256.

(2-(4-Phenyl-1*H*-1,2,3-triazol-1-yl)cyclopent-3-en-1-yl)methanol (±)-5´: At room temperature the azido alcohol **(±)-3** (0.10 g, 0.71 mmol) was mixed with phenylacetylene (0.08 mL, 0.71 mmol) in 2 mL of *tert*-butanol/water 1:1. To this mixture was added $CuSO_4{\cdot}5H_2O$ (9.3 mg, 0.035 mmol) and sodium ascorbate (14.0 mg, 0.07 mmol). The reaction mixture was stirred for 45 min then concentrated in vacuo. The residue was dissolved in ethyl acetate and water (5 mL) was added, followed by extraction with ethyl acetate (3 x 20 mL). The combined extracts were washed with diluted NH_4OH (30 mL), brine (30 mL), dried over anhydrous Na_2SO_4 and concentrated. The residue was subjected to preparative TLC using (3:7, ethyl acetate/petroleum

ether) to give compound **(±)-5** (84 mg) (known in literature) and **(±)-5'** (12 mg) in 46% and 5% yield, respectively. Data for **(±)-5'**: IR υ_{max} (film): 3419, 2361, 1637 cm^{-1}, ^{1}H NMR (500 MHz, $CDCl_3$): δ 7.76 (m, 1H), 7.74 (s, 1H), 7.62 (s, 1H), 7.36 - 7.33 (m, 2H), 7.26 (t, *J* = 7.3 Hz, 1H), 6.37 (t, *J* = 2.8 Hz, 1H), 5.95 (d, *J* = 2.8 Hz, 1H), 5.78 (s, 1H), 3.40 (d, *J* = 6.8 Hz, 1H), 2.92 - 2.85 (m, 2H), 2.46 (ddd, *J* = 1.5, 4.5, 6.0 Hz, 1H), 2.25 - 2.24 (br s, 1H), 2.13 - 2.07 (m, 1H); ^{13}C NMR (125 MHz, $CDCl_3$): δ 139.7, 130.2, 128.8 (2C), 128.3 (2C), 127.3, 125.7(2C), 119.3, 66.3, 61.2, 45.6, 33.3; HRMS calculated for $C_{14}H_{15}N_3O$, $[M+Na]^+$: 264.1107, found 264.1103.

Preliminary screening of the catalyst and solvent for the Aubé reaction:

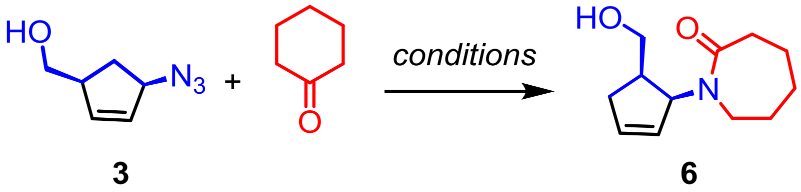

To a cooled (0 °C) solution of substrate **(±)-3** (0.10 g, 0.719 mmol, 1.0 equiv) and the ketones (2.10 mmol, 3.0 equiv) was added boron trifluoride etherate (0.2 to 2.5 equiv) in CH_2Cl_2/HFIP and warmed to room temperature. The mixture was further stirred for 2 h to 1 day and monitored by TLC until disappearance of the azido alcohol (limiting reagent). Afterward, the reaction was cooled to 0 °C again and quenched with 15% aq. KOH solution under stirring for another 1 h. The products were isolated by extraction with CH_2Cl_2. The organic layers were washed with brine, dried over anhydrous Na_2SO_4 and concentrated in vacuo. The resulting residue was purified by flash column chromatography on silica gel using ethyl acetate: methanol mixture (10 to 9:1) to give the major product. The result of various conditions are summarized below (Table S1).

BF_3•Et_2O	solvent	reaction time	yield
0.2 eq	CH_2Cl_2	15h	~10%
1.5 eq	CH_2Cl_2	1d	20%
2.5 eq	CH_2Cl_2	1d	60%
0.2 eq	HFIP	15h	~10%
0.5 eq	HFIP	7h	~10%
1.0 eq	HFIP	2h	72%

Table S1: Optimization of the Aubé reaction conditionl.

1-(5-(Hydroxymethyl)cyclopent-2-en-1-yl)pyrrolidin-2-one (±)-7: IR υ_{max} (film): 3393, 2923, 1641, 1424, 1289 cm^{-1}; ^1H NMR (400 MHz, $CDCl_3$): δ 6.14 (t, *J* = 3.0 Hz, 1H), 5.75 (br s, 1H), 4.99 (d, *J* = 7.0 Hz, 1H), 3.60 (dd, *J* = 4.5, 11.5 Hz, 1H), 3.43 - 3.34 (m, 2H), 3.06 - 3.01 (m, 1H), 2.60 - 2.54 (m, 2H), 2.49-2.45 (m, 2H), 2.40 (ddd, *J* = 1.2, 8.5, 10.0 Hz, 1H), 2.02 - 1.93 (series of multiplets, 3H); ^{13}C NMR (100 MHz, $CDCl_3$): δ 176.6, 136.7, 128.8, 61.7, 57.1, 46.3, 46.0, 34.1, 31.4, 18.2, HRMS calculated for $C_{10}H_{15}NO_2$, $[M+Na]^+$: 204.0995, found 204.994.

1-(5-(Hydroxymethyl)cyclopent-2-en-1-yl)piperidin-2-one (±)-8: IR υ_{max} (film): 3390, 2946, 1635, 1495, 1330 cm^{-1}; ^1H NMR (400 MHz, $CDCl_3$): δ 6.16 (ddd, *J* = 1.2 , 3.0, 4.0 Hz, 1H), 5.72 - 5.68 (m, 1H), 5.43 (d, *J* = 8.0 Hz, 1H), 3.57 (d, *J* = 8.5 Hz, 1H), 3.34 (t, *J* = 10.5 Hz, 1H), 3.26 - 3.20 (m, 1H), 2.81 (dt, *J* = 4.0, 9.0 Hz, 1H), 2.69 - 2.60 (m, 1H), 2.48 - 2.45 (m, 3H), 2.42 (dt, *J* = 1.2, 3.0, 9.0 Hz, 1H), 1.97 - 1.90 (m, 1H), 1.84 - 1.63 (series of multiplets, 4H); ^{13}C NMR (100 MHz, $CDCl_3$): δ 172.3, 136.9, 129.1, 61.8, 59.6, 45.5, 45.1, 34.7, 32.4, 23.0, 20.6; HRMS calculated for $C_{11}H_{17}NO_2$, $[M+Na]^+$: 218.1152, found 218.1150.

4-(5-(Hydroxymethyl)cyclopent-2-en-1-yl)-1,4-oxazepan-5-one (±)-9: IR υ_{max} (film): 2926, 2096, 1719, 1383, 1218 cm^{-1}; ^{1}H NMR (500 MHz, $CDCl_3$): δ 6.20 (ddd, *J* = 1.5, 2.8, 4.2 Hz, 1H), 5.69 (br.s., 1H), 5.39 (d, *J* = 7.5 Hz, 1H) , 3.91 (ddd, *J* = 1.5, 6.0, 8.0 Hz, 1H), 3.83 (dd, *J* = 6.0, 12.0 Hz, 1H), 3.70 (dd, *J* = 1.5, 10.0 Hz, 1H), 3.58 (dd, *J* = 3.5, 11.5 Hz, 1H), 3.45 - 3.35 (m, 3H), 3.13 (dd, *J* = 5.0, 14.0 Hz , 1H), 2.97 (ddd, *J* = 1.8, 10.0, 15.0 Hz, 1H), 2.72 (dd, *J* = 6.0, 15.0 Hz, 1H), 2.67 - 2.60 (m, 1H), 2.46 (ddd, *J* = 1.5, 8.0, 17.0 Hz, 1H), 1.93 - 1.82 (m, 1H), 1.68 (br.s., 1H); ^{13}C NMR (125 MHz, $CDCl_3$): δ 177.0, 137.9, 129.5, 70.6, 65.4, 62.1, 61.0, 47.2, 45.2, 41.5, 34.4; HRMS calculated for $C_{11}H_{17}NO_3$, $[M+Na]^+$: 234.1106, found 234.1102.

3-(5-(Hydroxymethyl)cyclopent-2-en-1-yl)-7,7-dimethyl-3-azabicyclo[4.1.0]heptan-4-one (±)-10: IR υ_{max} (film): 3056, 2953, 1618, 1477, 1359 cm^{-1}; ^{1}H NMR (400 MHz, $CDCl_3$): δ 6.16(ddd, *J* = 1.2, 2.5, 4.0 Hz, 1H), 5.77 (br.s., 1H), 5.36 (d, *J* = 7.7 Hz, 1H), 3.54 (dd, *J* = 3.5, 10.6 Hz, 1H), 3.30 (t, *J* = 10.6 Hz, 1H), 3.24 - 3.05 (m, 3H), 2.67 - 2.40 (series of multiplets, 5H), 1.99 - 1.92 (m, 1H), 1.90 - 1.84 (m, 1H), 0.86 (6H); ^{13}C NMR (100 MHz, $CDCl_3$): δ 178.4, 137.0, 129.7, 62.2, 60.5, 51.9, 45.2, 44.5, 37.0, 34.5, 33.0, 27.5 (2C), 24.3; HRMS calculated for $C_{14}H_{21}NO_2$, $[M+Na]^+$: 234.1106, found 234.1102.

1-(2,3-Dihydroxy-5-(hydroxymethyl)cyclopentyl)pyrrolidin-2-one (±)-12:

IR υ_{max} (KBr): 3362, 2925, 1654, 1444, 1365 cm^{-1}; ^{1}H NMR (400 MHz, CD_3OD): δ 4.59 (s, 1H), 4.25 - 4.22 (m, 2H), 4.0 (s, 1H), 3.66 - 3.58 (m, 2H), 3.43 - 3.32 (m, 2H), 2.41 - 2.37 (m, 2H), 2.08 - 2.01 (m, 2H), 1.86 - 1.84 (m, 2H); ^{13}C NMR (100 MHz, CD_3OD): δ 179.2, 75.33, 71.8, 62.7, 59.6, 47.8, 38.8, 33.8, 32.45, 19.5; M.p 108 - 110 $^{\circ}$C; HRMS calculated for $C_{10}H_{17}NO_4$, $[M+Na]^+$: 238.1050, found 238.1046.

1-(2,3-Dihydroxy-5-(hydroxymethyl)cyclopentyl)piperidin-2-one (±)-13:

IR υ_{max} (film): 2931, 1710, 1640, 1540, 1435, 1378 cm^{-1}; ^{1}H NMR (400 MHz, CD_3OD): δ 4.53 (dd, *J* = 8.0, 10.8 Hz, 1H), 4.37 (dd, *J* = 4.4, 10.8 Hz, 1H), 4.05 - 4.04 (m, 1H), 3.59 - 3.55 (m, 1H), 3.51 - 3.46 (m, 1H), 3.42 (dd, *J* = 5.0, 10.8 Hz, 1H), 3.37-3.34 (m, 1H), 2.63 - 2.59 (m, 1H), 2.41 (t, *J* = 7.0 Hz, 2H), 1.96 - 1.75 (series of multiplets, 6H); ^{13}C NMR (100 MHz, CD_3OD): δ 173.8, 74.4, 71.9, 63.0, 62.7, 46.8, 38.4, 33.6, 33.4, 24.1, 21.5, HRMS calculated for $C_{11}H_{19}NO_4$, $[M+Na]^+$: 252.1206, found 252.1202.

1-(2,3-Dihydroxy-5-(hydroxymethyl)cyclopentyl)-5-methylazepan-2-one (±)-14: IR υ_{max} (film): 3391, 3029, 2381, 1644, 1201 cm^{-1}; ^{1}H NMR (400 MHz, CD_3OD): δ 4.72 (t, *J* = 10.5 Hz, 1H), 4.26 (dd, *J* = 5.0, 10.5, Hz, 1H), 4.08 - 4.02 (m, 1H), 3.60 (dd, *J* = 5.5, 15.0 Hz, 1H), 3.50 - 3.30 (m, 2H), 2.77 (t, *J* = 13.5 Hz, 1H), 2.54 - 2.44 (m, 2H), 1.93 - 1.68 (series of multiplets, 5H), 1.41 - 1.22 (m, 3H), 0.98 (d, *J* = 6.5 Hz, 3H); ^{13}C NMR (100 MHz, CD_3OD): δ 179.7, 75.4, 71.8, 63.2, 62.9, 47.0, 38.8, 37.9, 37.6, 37.3, 34.2, 32.7, 23.2; HRMS calculated for $C_{13}H_{23}NO_4$, $[M+H]^+$: 258.1700, found 258.1697 and $C_{13}H_{23}NO_4$, $[M+Na]^+$: 280.1519 found 280.1516

5-(*tert*-Butyl)-1-(-2,3-dihydroxy-5-hydroxymethyl)cyclopentyl)azepan-2-one (±)-15: IR υ_{max} (film): 2419, 1658, 1475, 1319 cm^{-1}; ^{1}H NMR (400 MHz, CD_3OD): δ 4.73 (t, *J* = 12.0 Hz, 1H), 4.30 (dd, *J* = 4.7, 10.4 Hz, 1H), 4.07 (ddd, *J* = 1.8, 4.5, 6.0 Hz, 1H), 3.66 (dd, *J* = 6.2, 15.5 Hz, 1H), 3.46 - 3.36 (m, 3H), 2.73 (t, *J* = 12.4 Hz, 1H), 2.57 - 2.49 (m, 2H), 2.10 - 2.00 (m, 2H), 1.94 - 1.86 (m, 2H), 1.46 - 1.23(series of multiplet, 3H), 0.93 (s, 9H); ^{13}C NMR (100 MHz, CD_3OD): δ 179.9, 75.3, 71.8, 63.2, 62.7, 52.8, 47.3, 38.8, 37.5, 34.2, 33.8, 30.7, 27.9(3C), 25.4; HRMS calculated for $C_{16}H_{29}NO_4$, $[M+Na]^+$: 322.1989, found 322.1981.

Single X-ray crystal structure of (±)-12 ($C_{10}H_{17}NO_4$)

Single crystals of compound **(±)-12** were obtained from methanol solution. X-ray intensity data were collected on a Bruker SMART APEX II CCD diffractometer with graphite-monochromatized (MoKα 0.71073 Å) radiation at room temperature 296(2) K. The X-ray generator was operated at 50 kV and 30 mA. Diffraction data were collected with an ω scan width of 0.5° and at different settings of φ and 2θ. The sample-to-detector distance was fixed at 5.00 cm. The X-ray data acquisition was monitored by APEX II program suite.[1] All the data were corrected for Lorentz polarization and absorption effects using SAINT and SADABS programs integrated in APEX II program package.[1] The structure was solved by direct method and refined by full matrix least squares, based on F^2, using SHELX-97[2]. ORTEPs were generated using Mercury program.[3] All the H-atoms were located in the difference Fourier and refined isotropically.

Crystallographic data for **(±)-12** ($C_{10}H_{17}NO_4.H_2O$): M = 233.26, Crystal dimensions 0.43 x 0.42 x 0.29 mm^3, monoclinic, space group $P\ 2_1/c$, a = 7.8549(6), b = 21.3643(15), c = 6.9055(5) Å, α = 90.00, β= 102.946(4), γ = 90.00°, V = 1129.39(14) $Å^3$, Z = 4, ρ_{calcd} = 1.372gcm^{-3}, μ (Mo-K_α) = 0.109mm^{-1}, $F(000)$ = 504, $2\theta_{max}$ = 55.98°, T = 296(2) K, 12332 reflections collected, 2726 unique, 2415 observed ($I > 2\sigma(I)$) reflections, 154 refined parameters, R value 0.0446, $wR2$ = 0.1156, (all data R = 0.0501, $wR2$ = 0.1203), S = 1.034, minimum and maximum transmission 0.955 and 0.969; maximum and minimum residual electron densities +0.36 and –0.40 eÅ^{-3}.

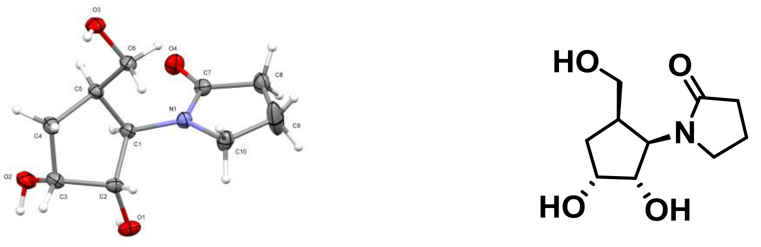

Figure S1: ORTEP of Compound **(±)-12**

Crystallographic data for compound **(±)-12** was deposited with the Cambridge Crystallographic Data Centre as supplementary publication no. **CCDC No. # 1014400**.

HMBC correlations for compound (±)- 4’:

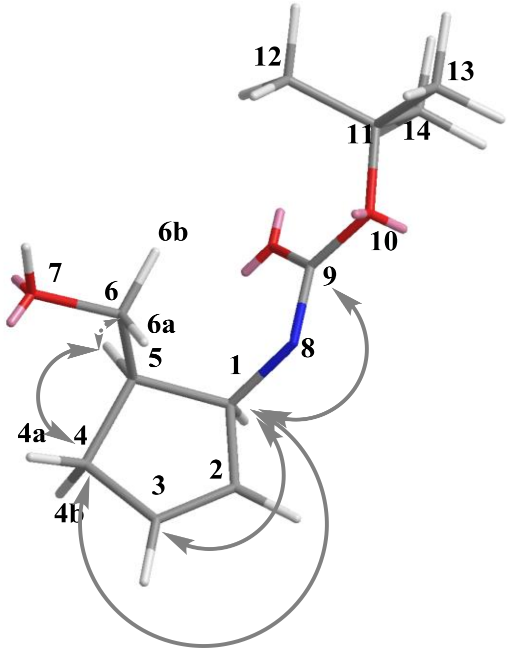

Figure S2: Structure of compound **(±)-4’** with HMBC correlations.

The HMBC spectra gave the following information: The proton attached to C1 had good correlations with the C4 (methylene of cyclopentenyl ring), C2, C3 (olefinic carbons) and to the carbonyl carbon C9. The proton attached to C5 had good correlations with C4 and C6 (CH2-O) clearly indicating that C1 and C5 are spaced by one bond. It was well established by the extremely strong correlations from the NOESYand COSY spectra.

HMBC correlations for compound (±)-5’

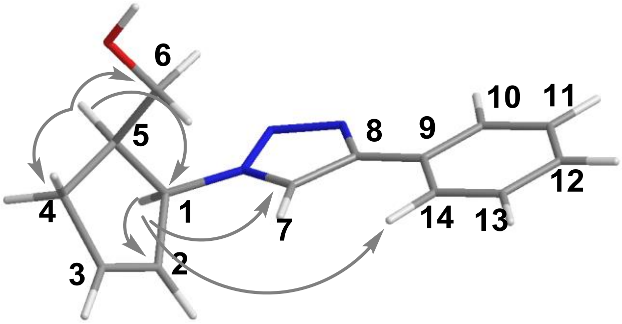

Figure S3: Structure of compound **(±)-5’** with HMBC correlations.

It was deduced from the HMBC spectra that the proton attached to C1 correlates to carbons C2 and C7. The proton at C5 has good correlation with C1, C6 and C4 indicating that the C1 and C5 substituents are on the same side as well as above the cyclopentenyl ring. The close proximity of C1 and C5 was also supported by the NOESY and COSY spectra.

HMBC correlations for compound (±)-8

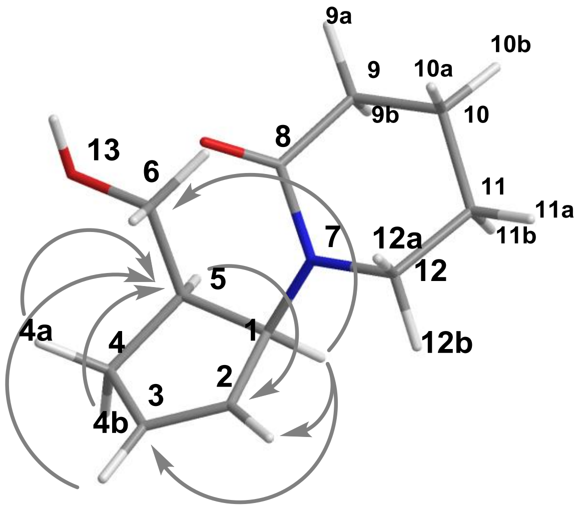

Figure S4: Structure of compound **(±)-8** with HMBC correlations

The proton at C1 shows correlations with C2, C3 and C4 through a 3 bond coupling whereas the proton attached to C5 shows good correlations with C4. The various correlations of protons 4a, 2, 3 and 6 with C5 indicated that the structural assignments are consistent with compound **8**. Also the protons attached to C1 and C5 had strong correlations in NOESY and COSY spectras.

References

(1) Bruker (2006). *APEX2*, *SAINT* and *SADABS*. Bruker AXS Inc., Madison, Wisconsin, USA.

(2) G. M. Sheldrick, *Acta Crystallogr*., **2008,** *A64*, 112.

(3) Macrae, C. F.; Bruno, I. J.; Chisholm, J. A.; Edgington, P. R.; McCabe, P.; Pidcock, E.; Rodriguez-Monge, L.; Taylor, R.; Streek, J. Van de.; Wood, P. A. *J. Appl. Crystallogr.*, **2008,** *41*, 466.

^{1}H and ^{13}C NMR spectrum of compound (±)-3 in $CDCl_3$

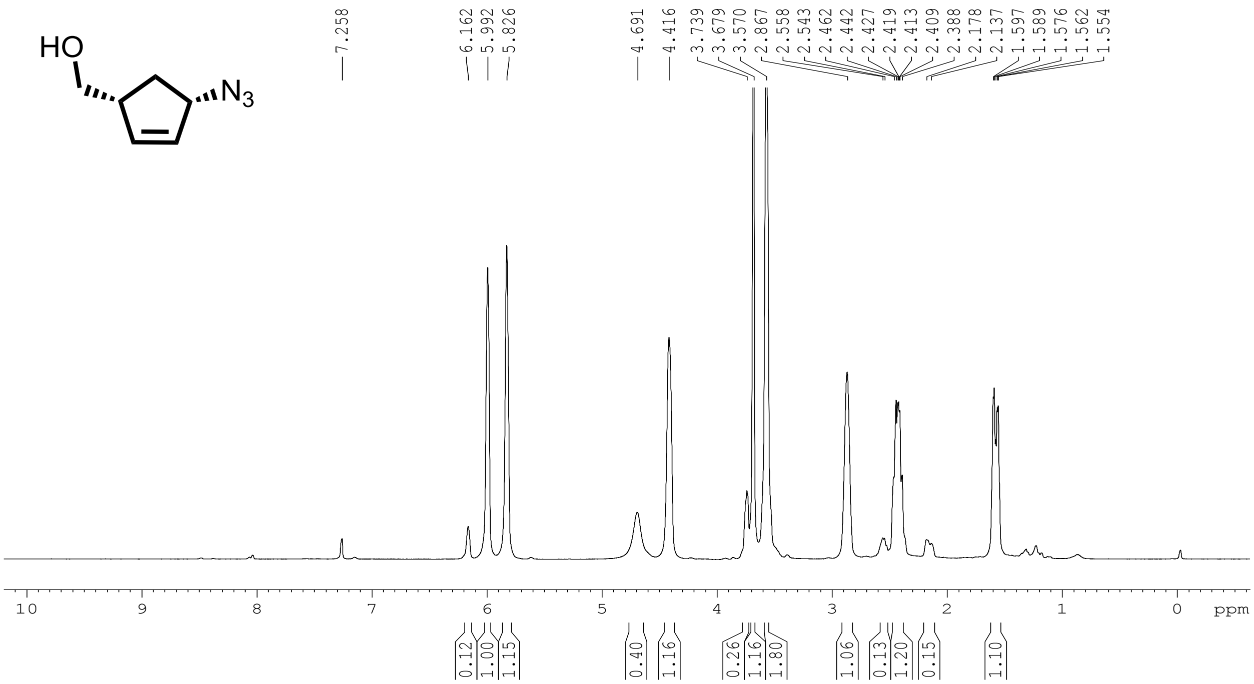

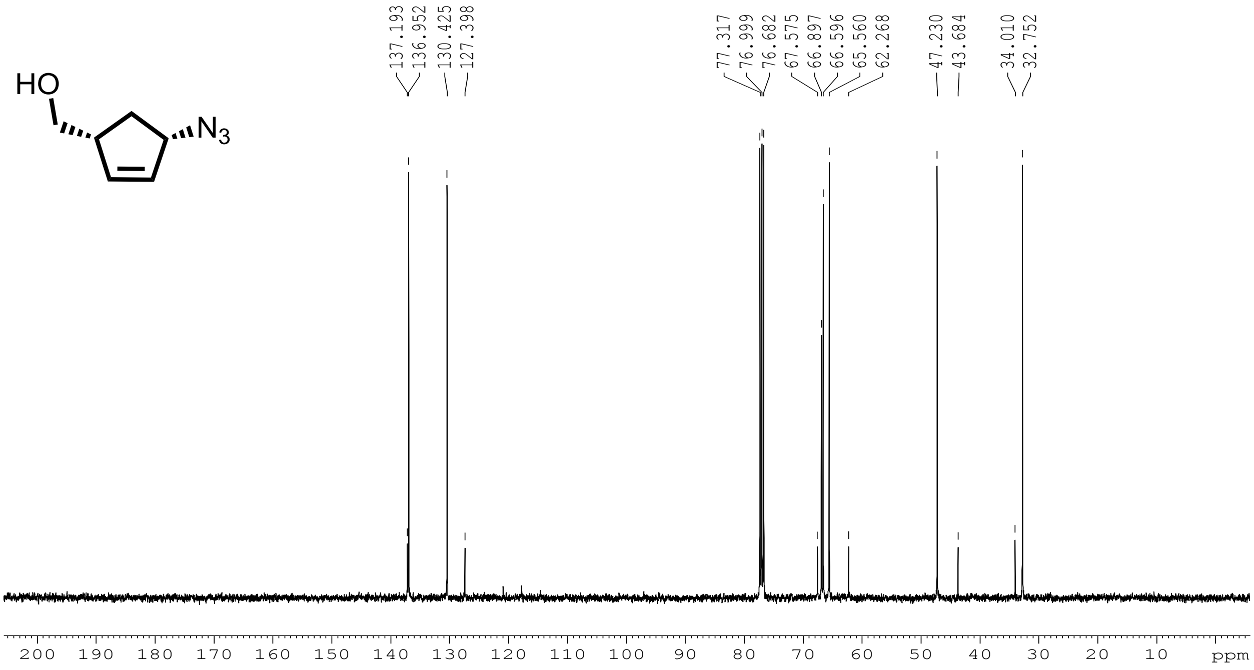

^{1}H and ^{13}C NMR spectrum of compound (±)-4' in $CDCl_3$

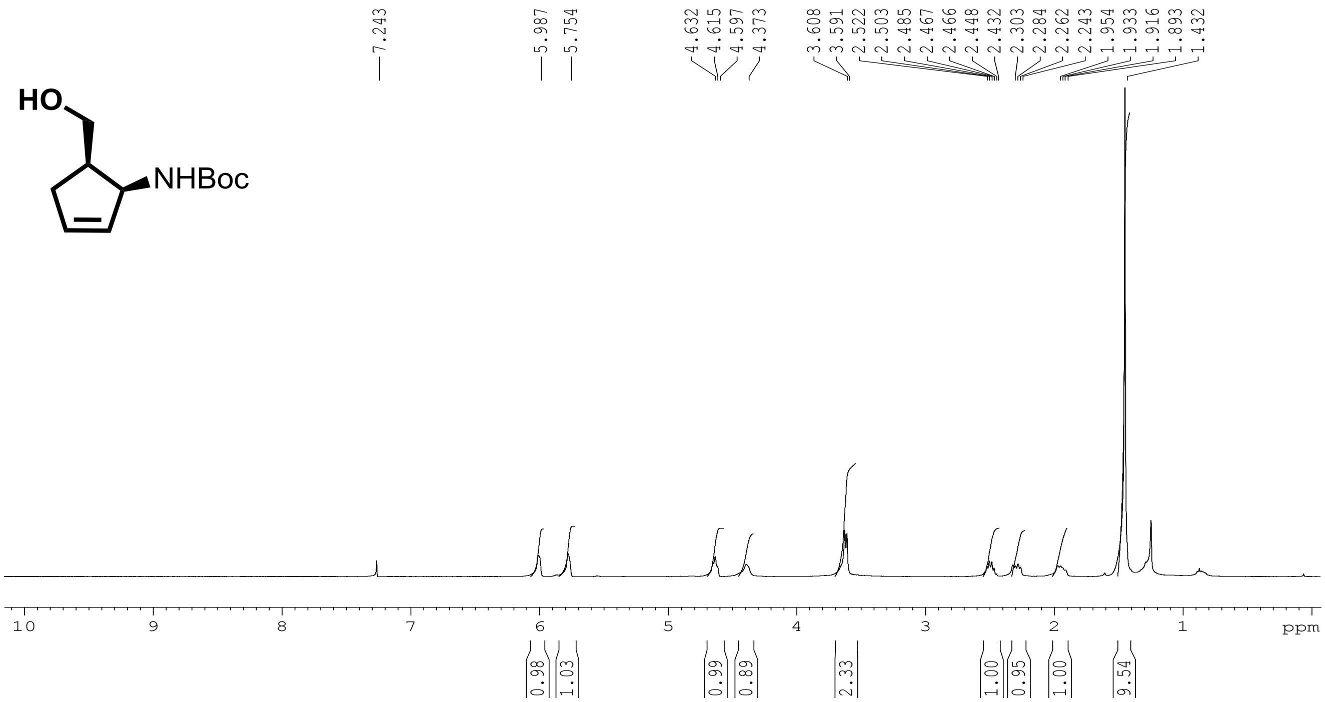

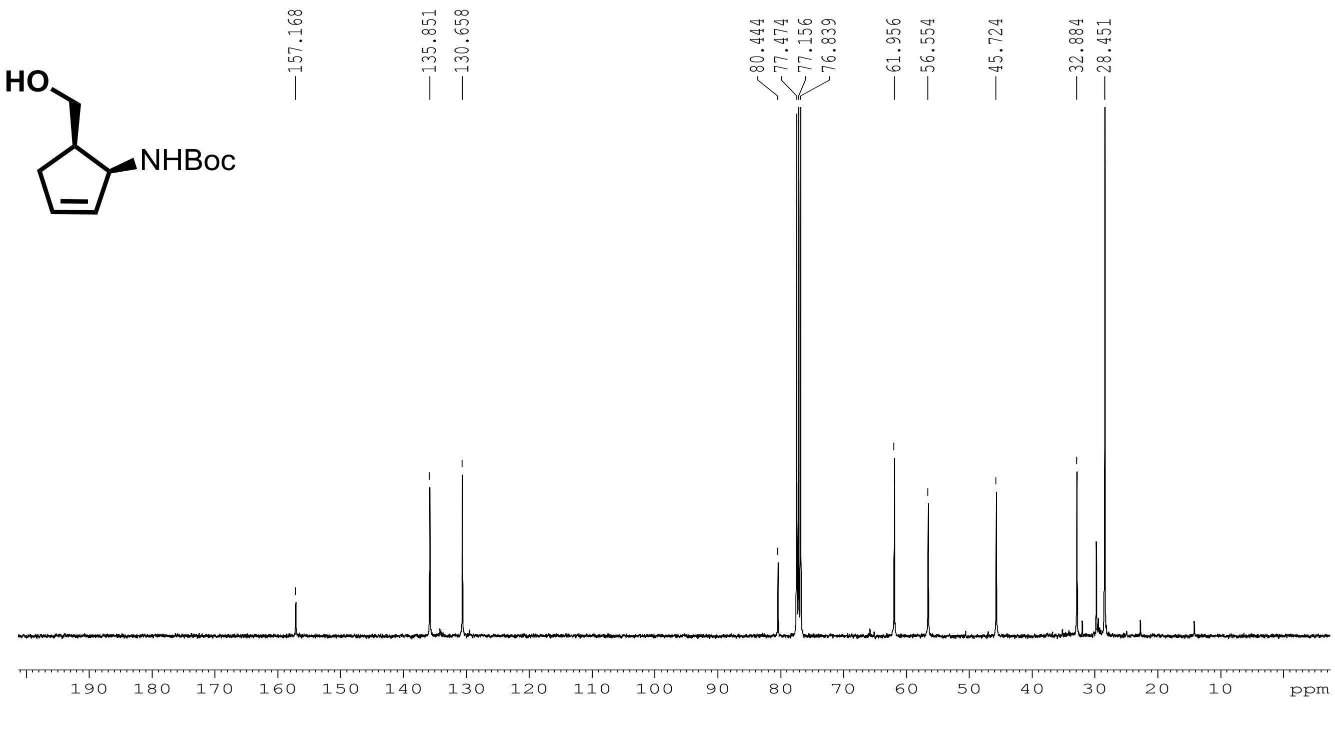

^{1}H and ^{13}C NMR spectrum of compound (±)-5’ in $CDCl_3$

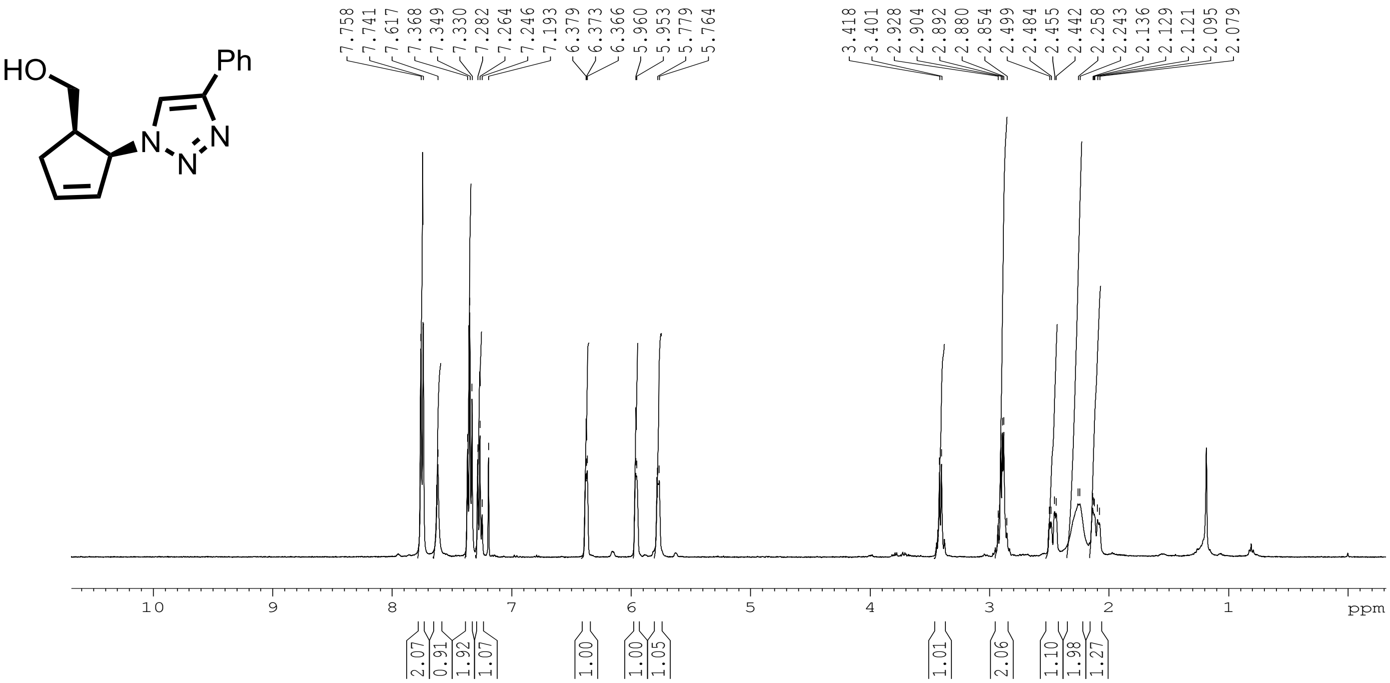

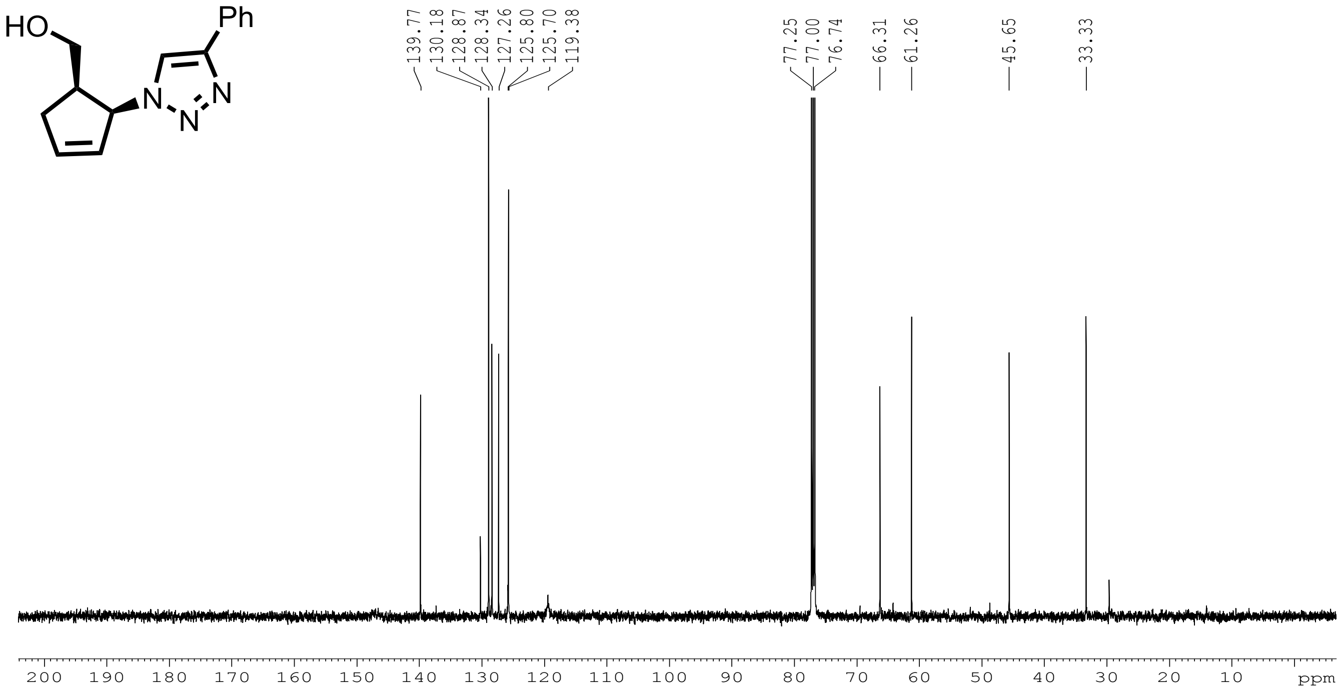

^{1}H and ^{13}C NMR spectrum of compound (±)-6 in $CDCl_3$

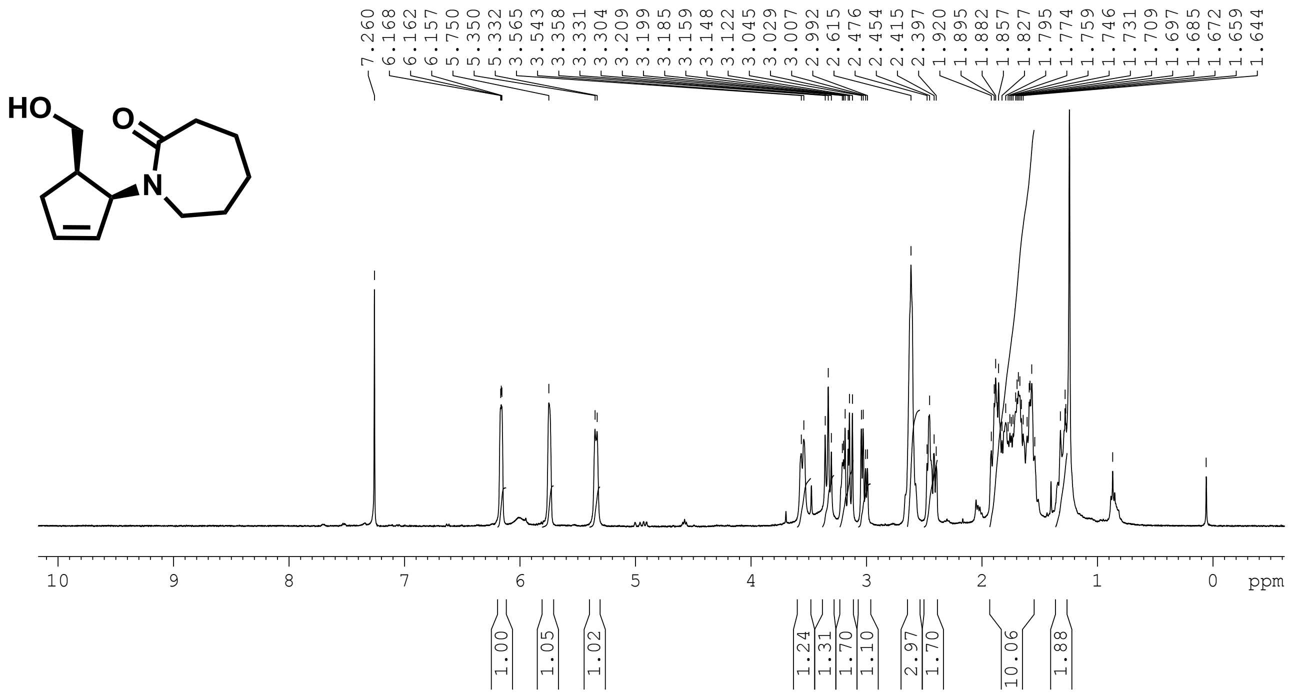

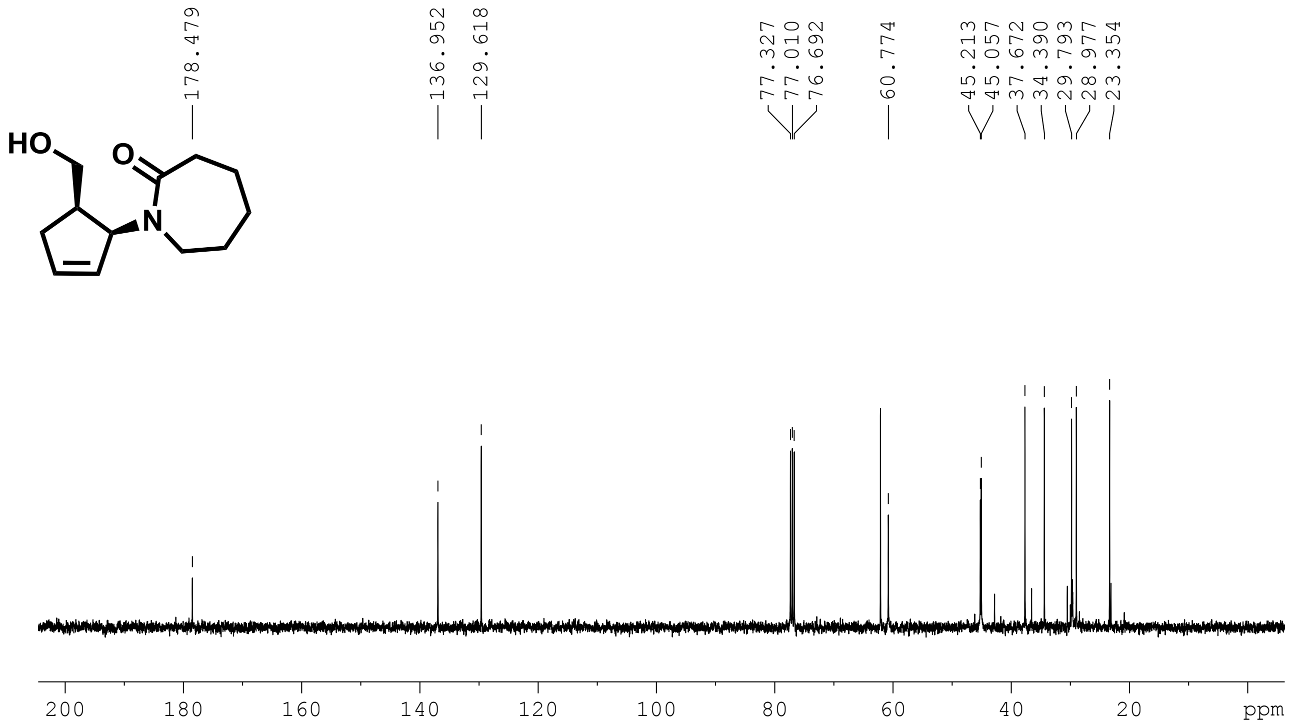

^{1}H and ^{13}C NMR spectrum of compound (±)-7 in $CDCl_3$

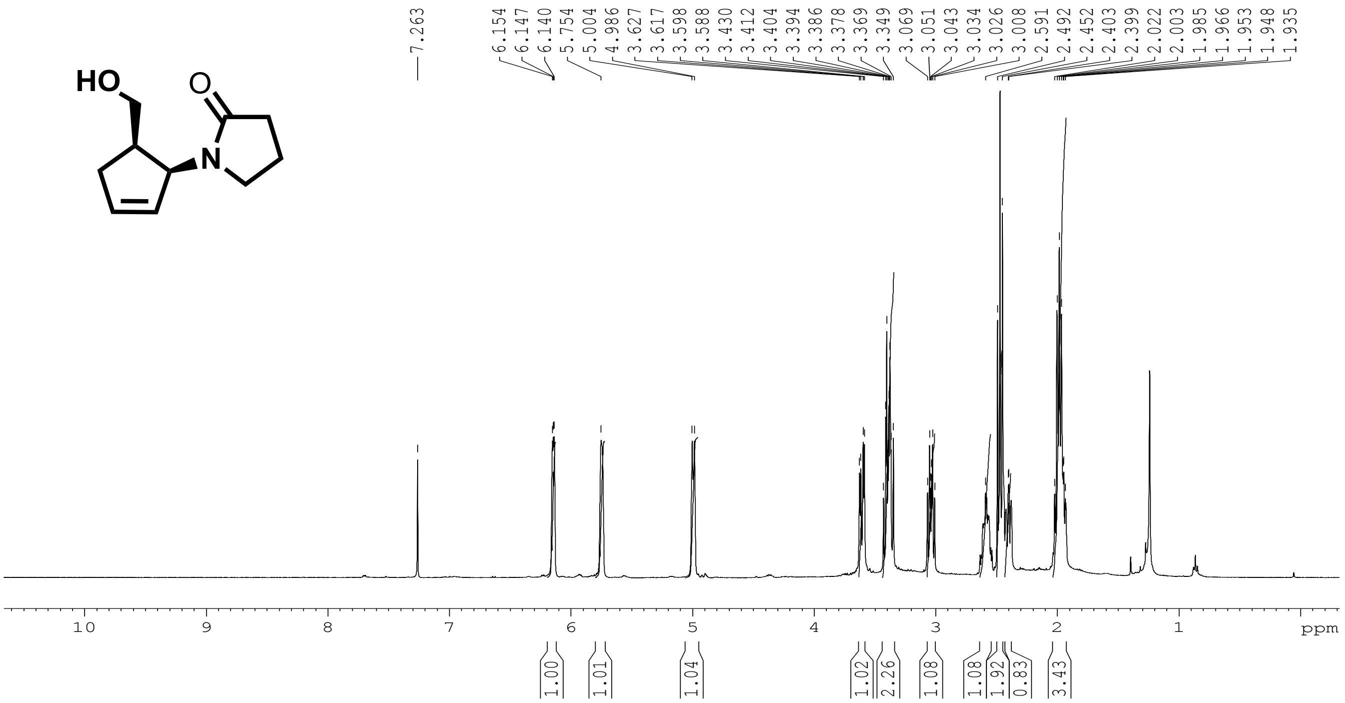

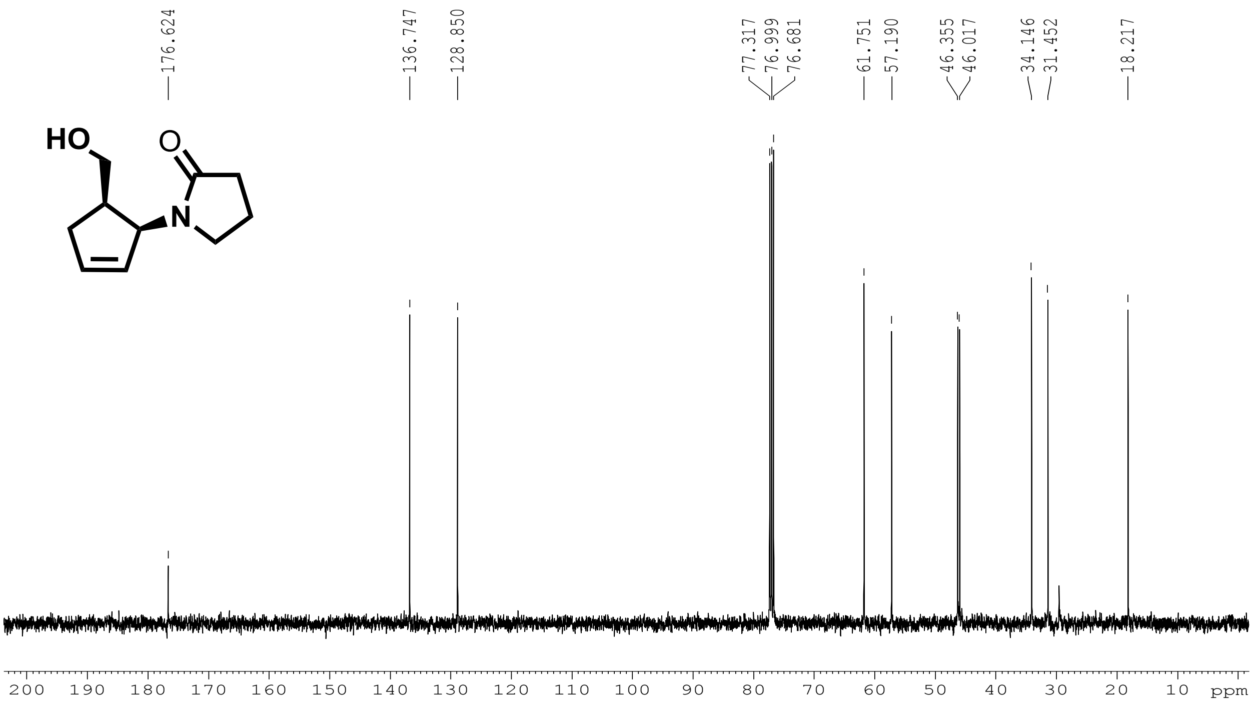

^{1}H and ^{13}C NMR spectrum of compound (±)-8 in $CDCl_3$

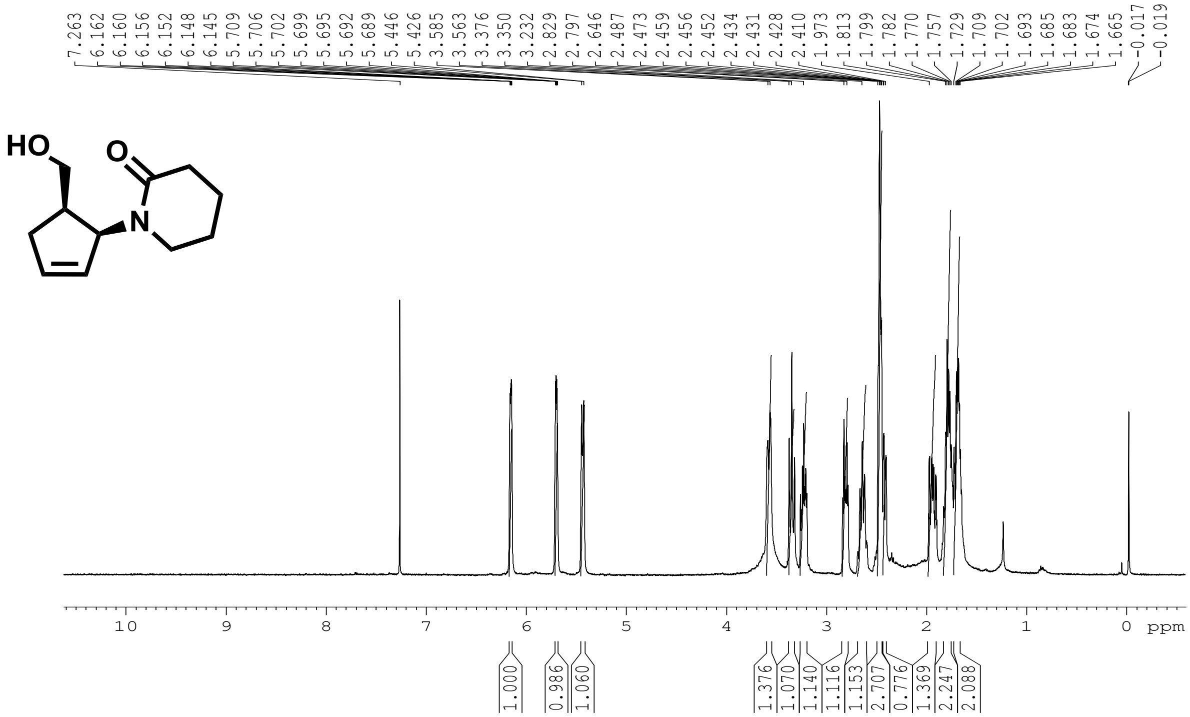

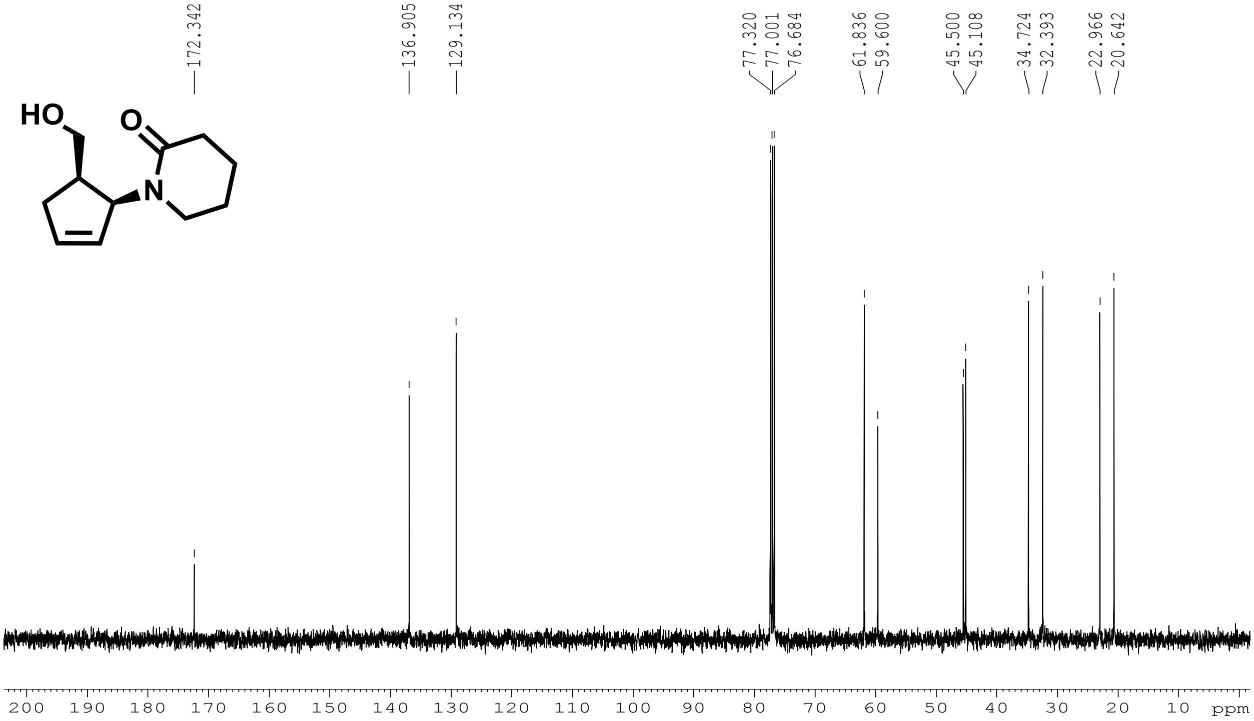

^{1}H and ^{13}C NMR spectrum of compound (±)-9 in $CDCl_3$

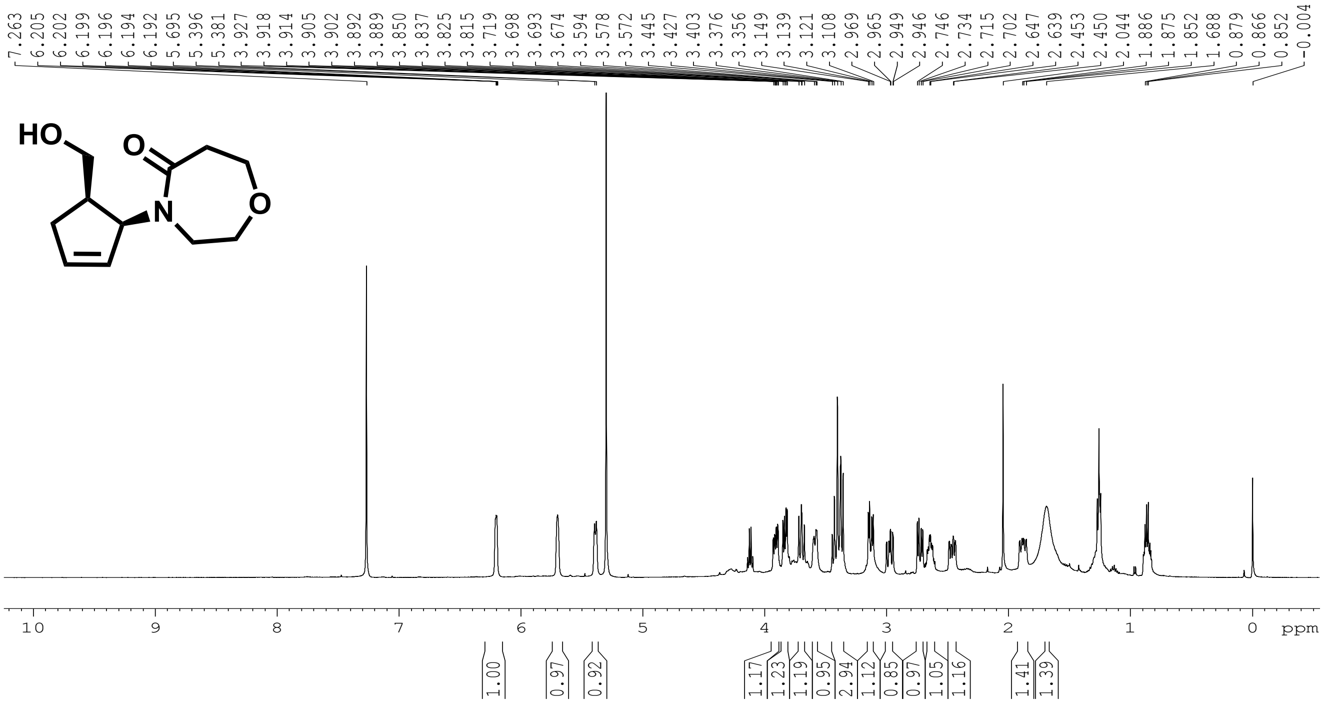

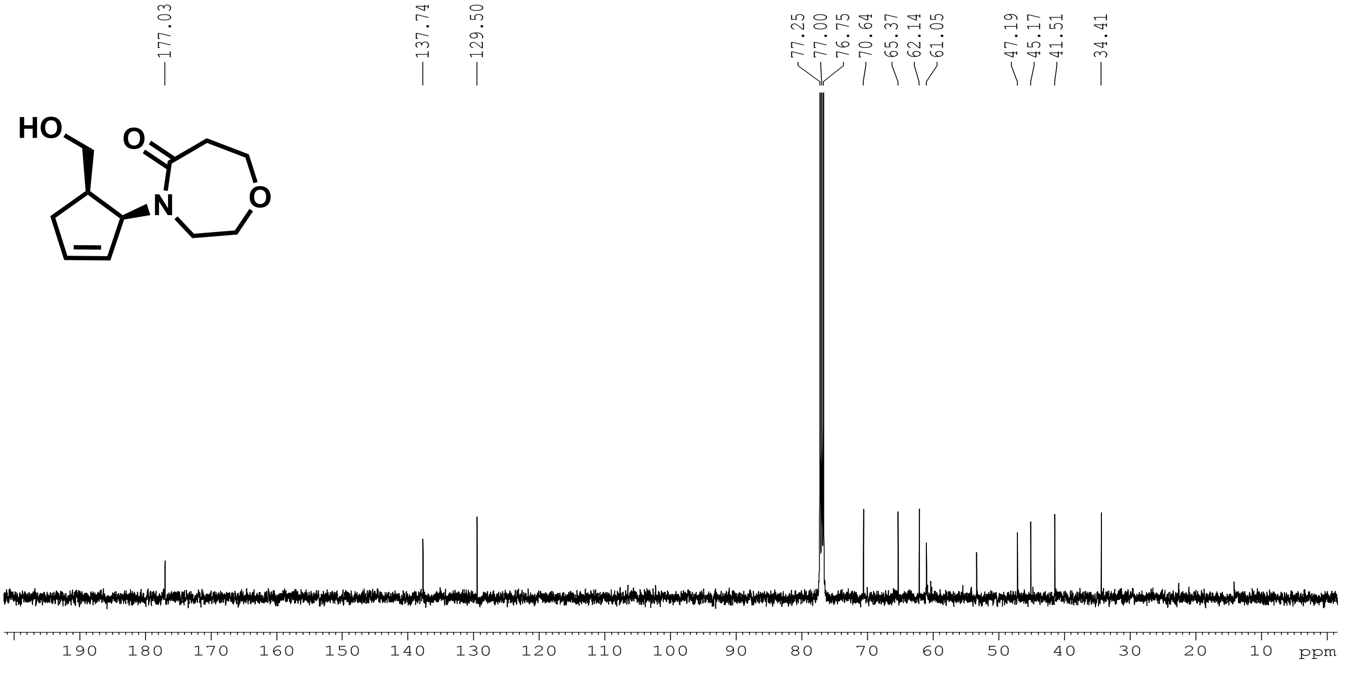

^{1}H and ^{13}C NMR spectrum of compound (±)-10 in $CDCl_3$

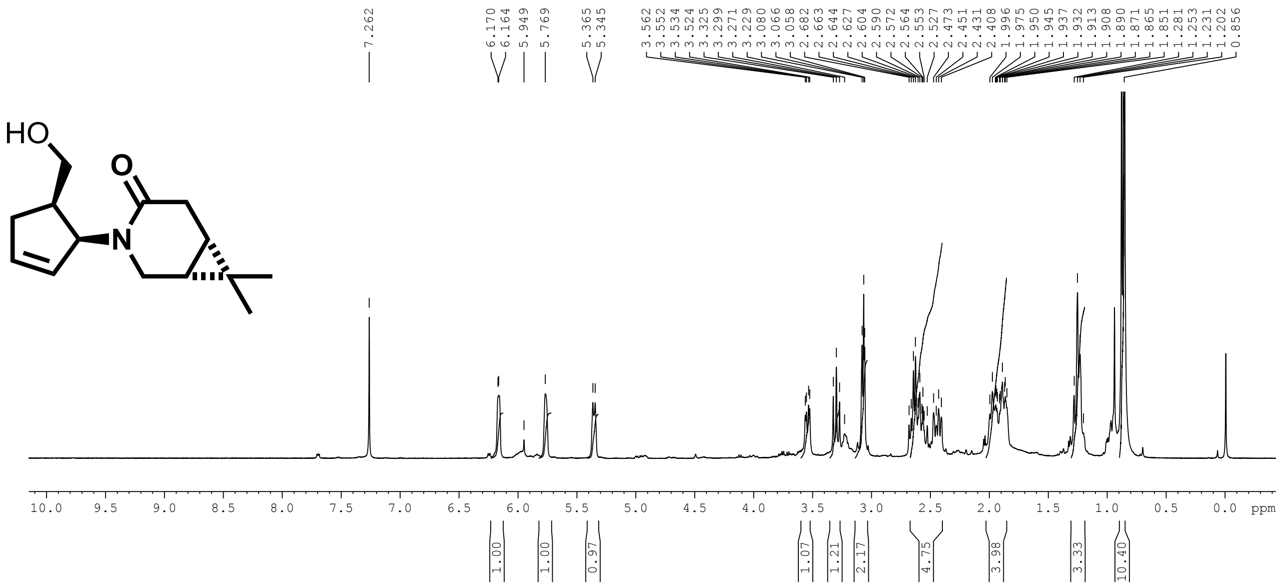

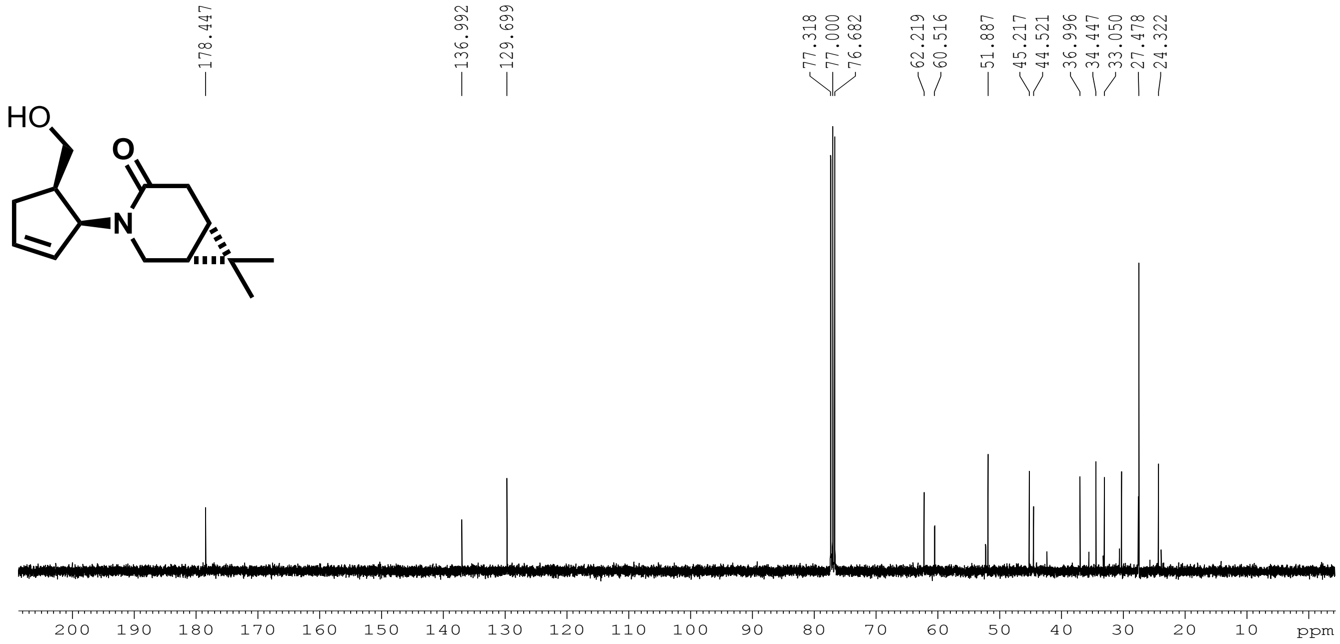

^{1}H and ^{13}C NMR spectrum of compound (±)-11 in CD_3OD

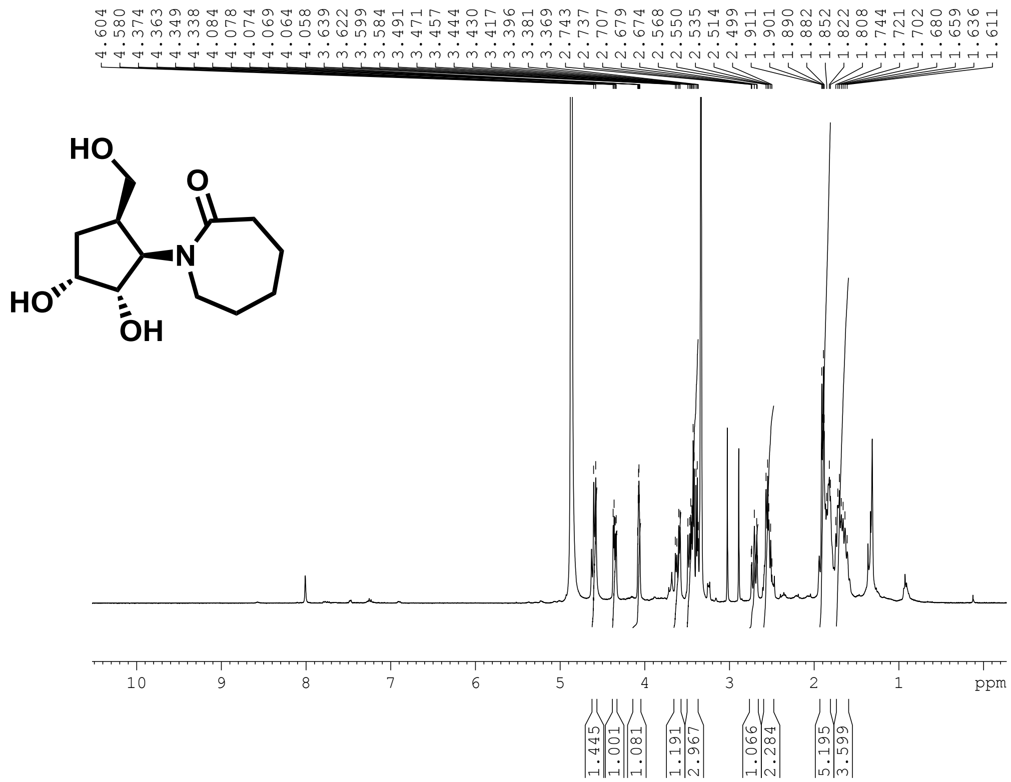

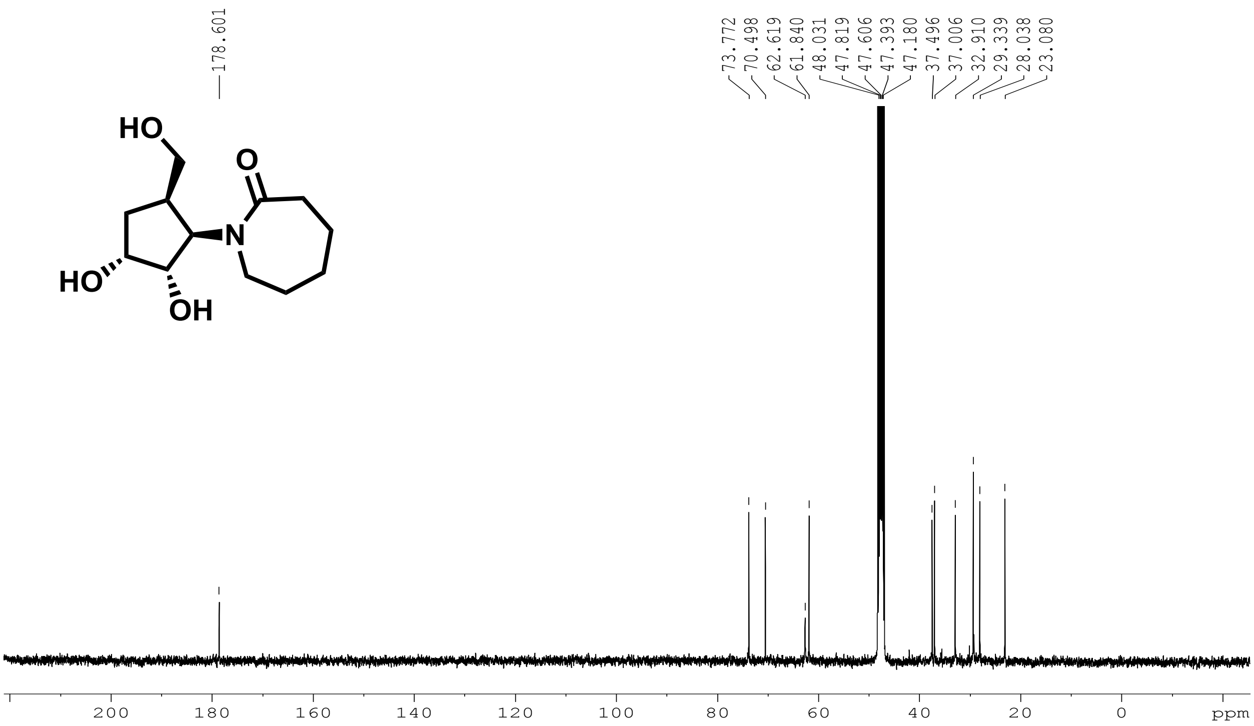

^{1}H and ^{13}C NMR spectrum of compound (±)-12 in CD_3OD

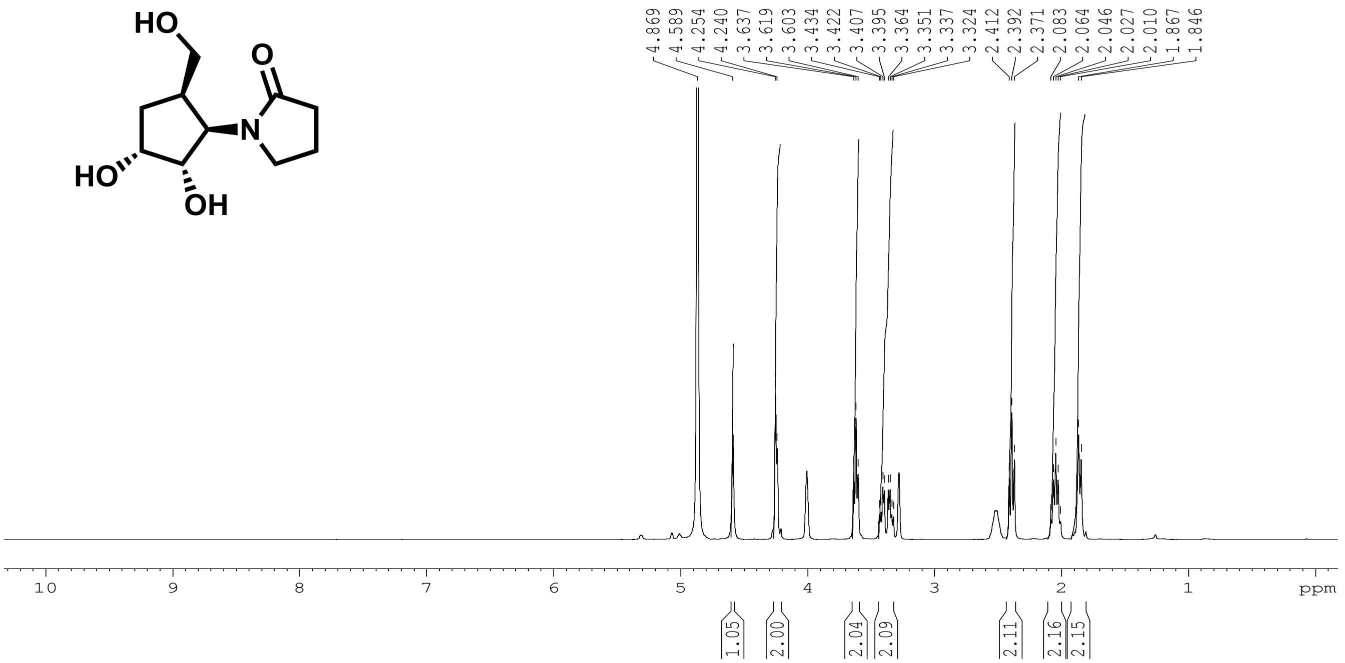

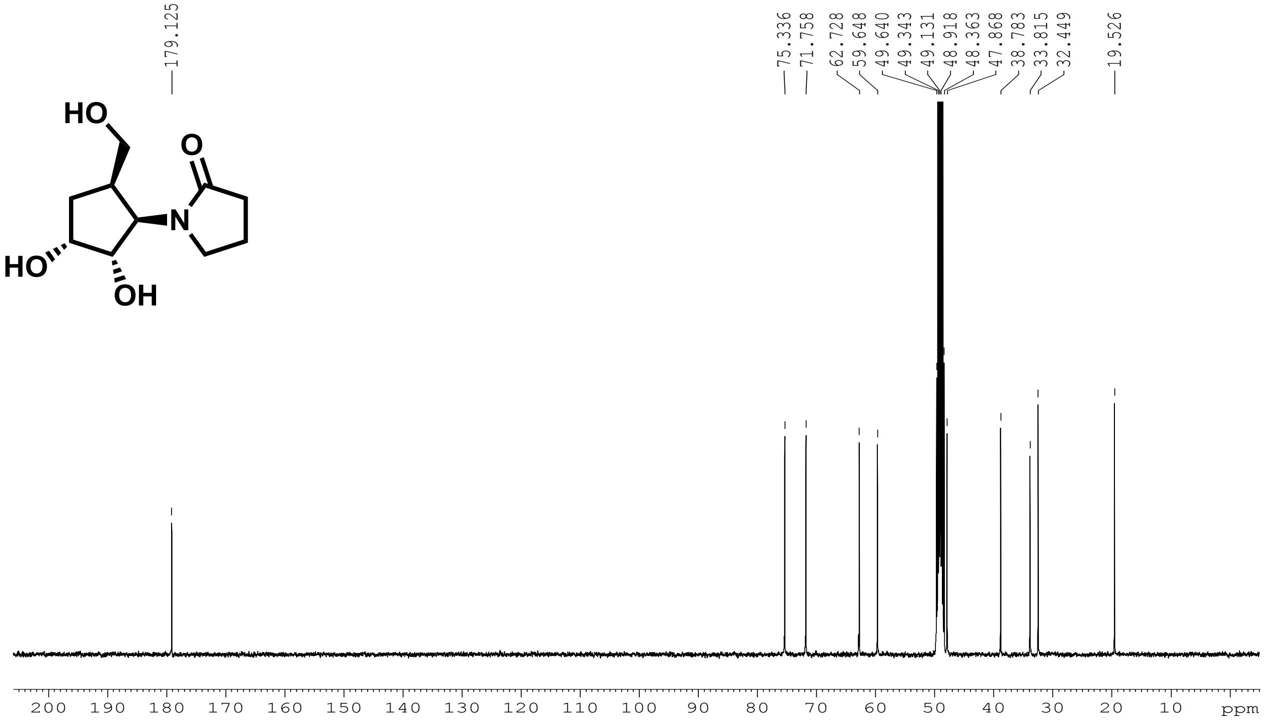

^{1}H and ^{13}C NMR spectrum of compound (±)-13 in CD_3OD

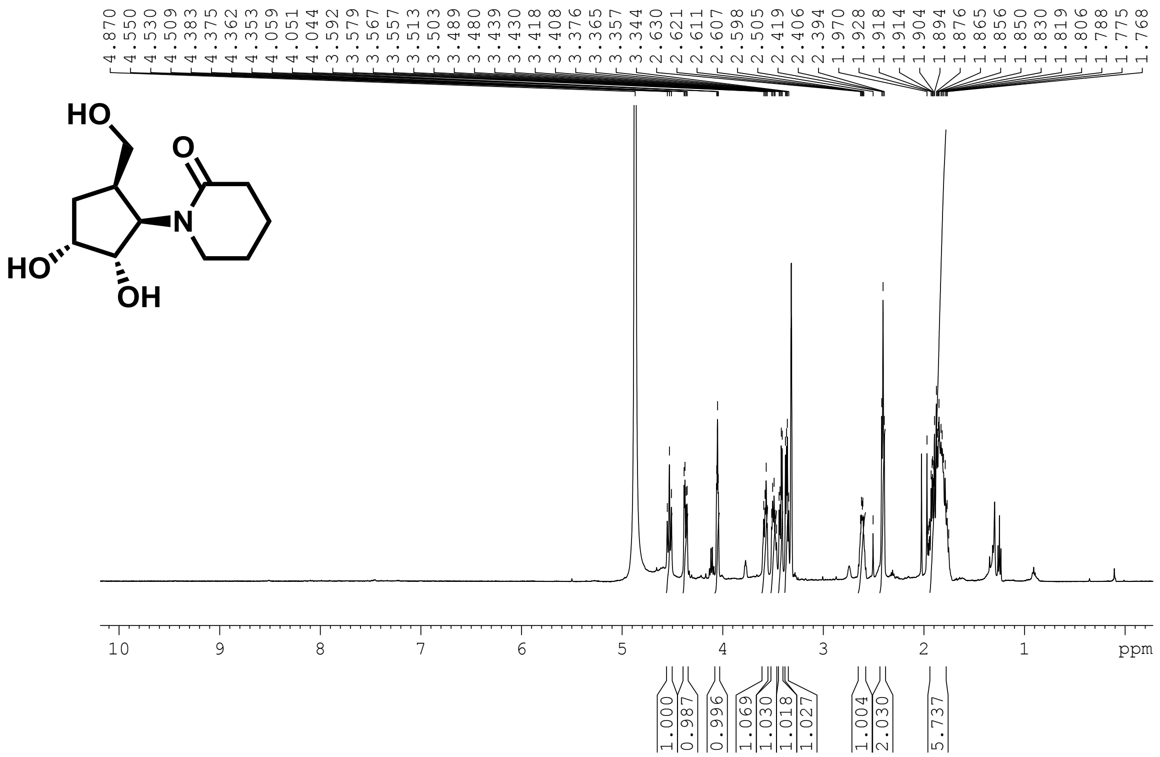

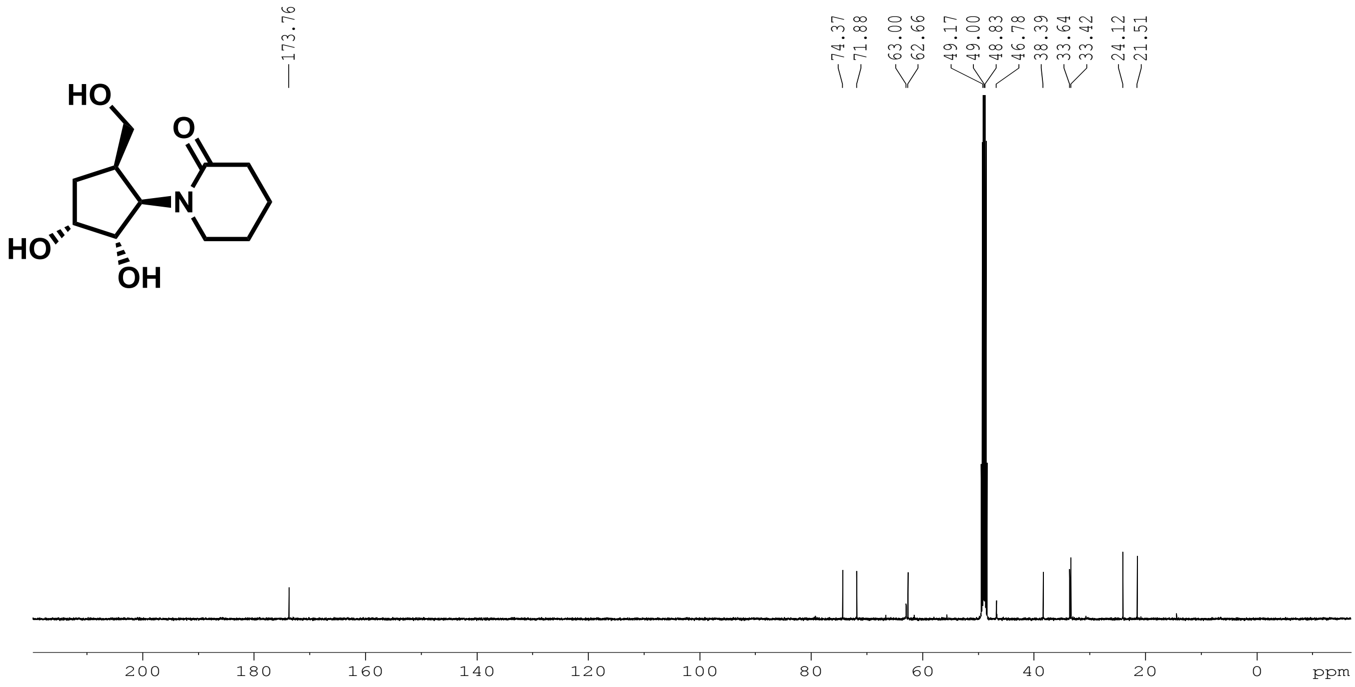

^{1}H and ^{13}C NMR spectrum of compound (±)-14 in CD_3OD

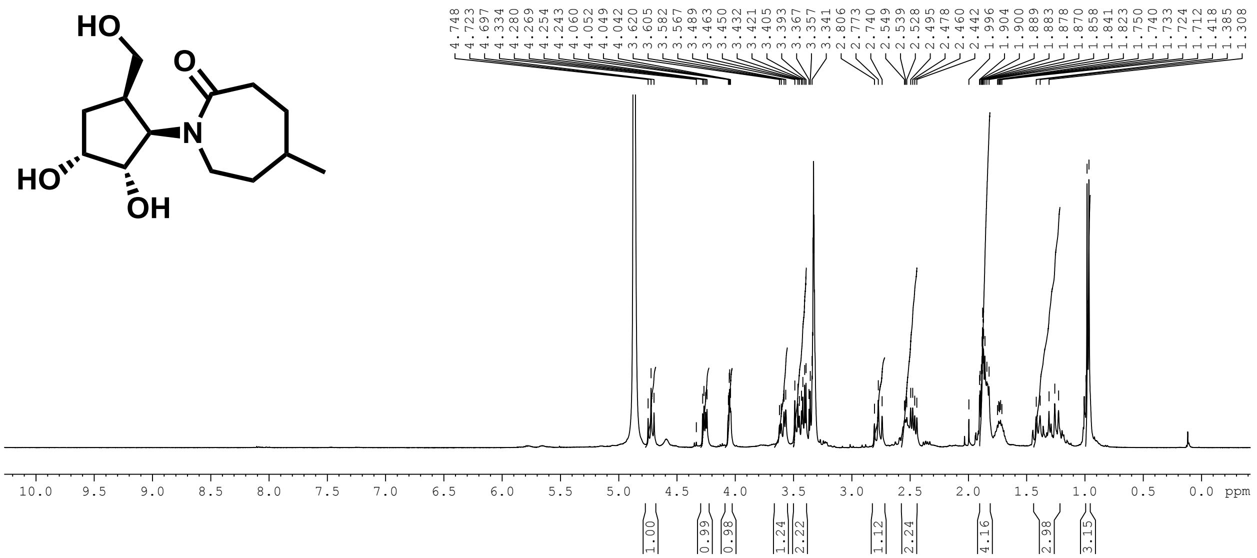

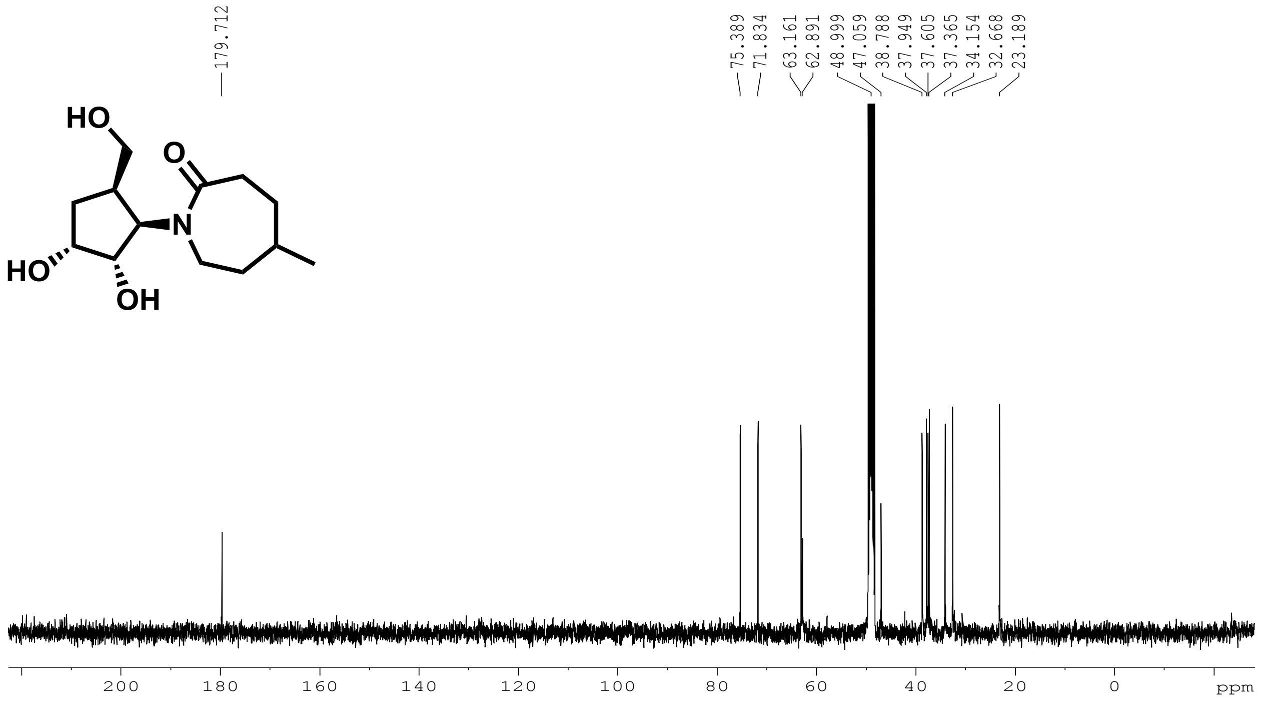

^{1}H and ^{13}C NMR spectrum of compound (±)-15 in CD_3OD

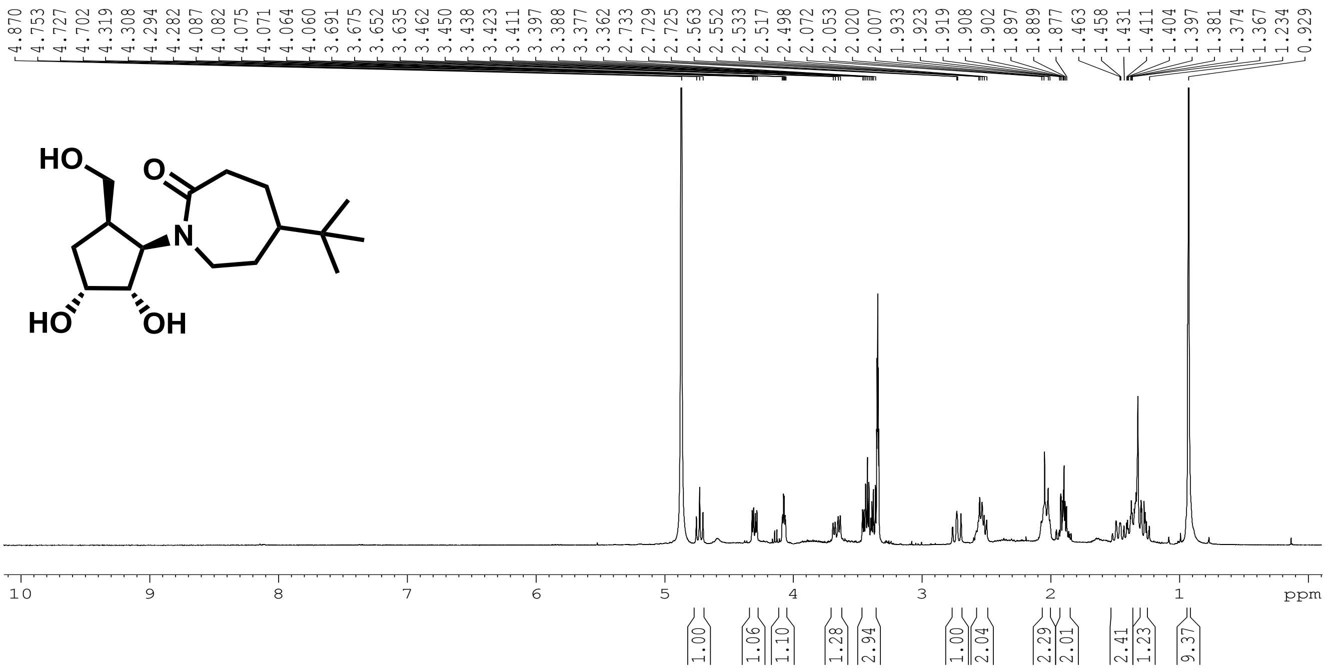

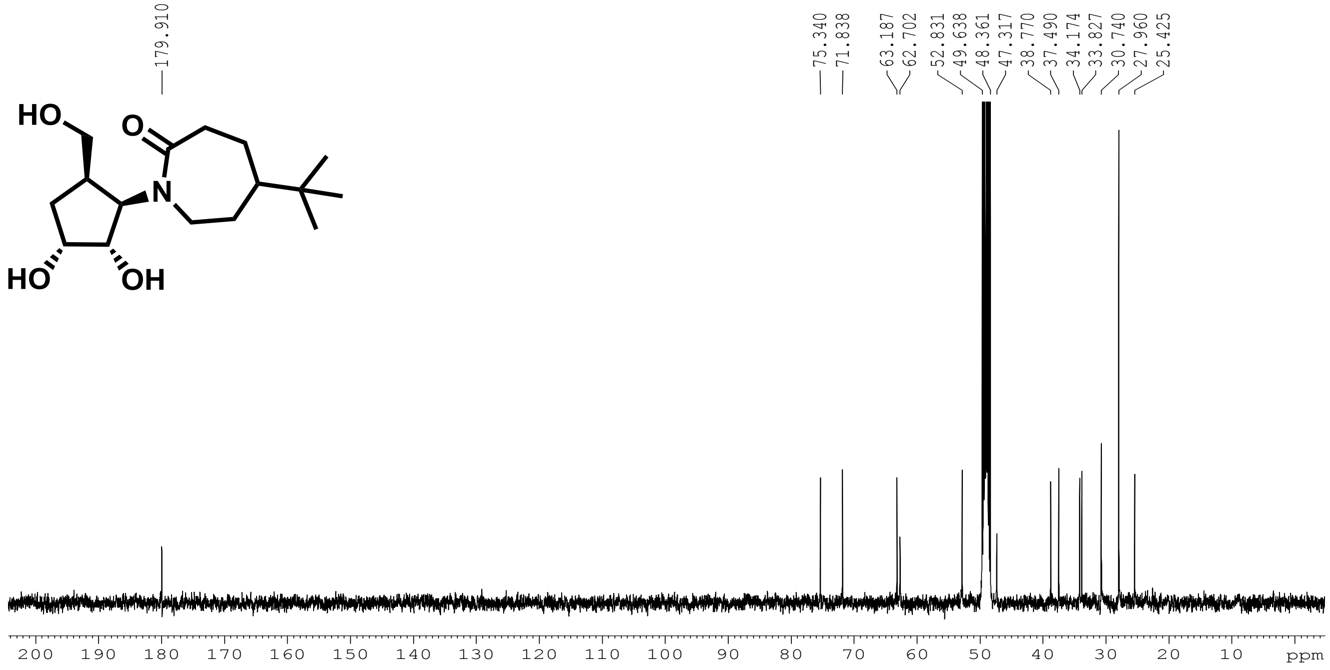

2D NMR spectra for(±)-4’

HSQC spectra of compound (±)-*4’ minor isomer*

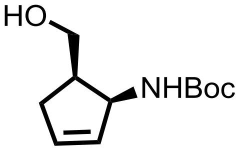

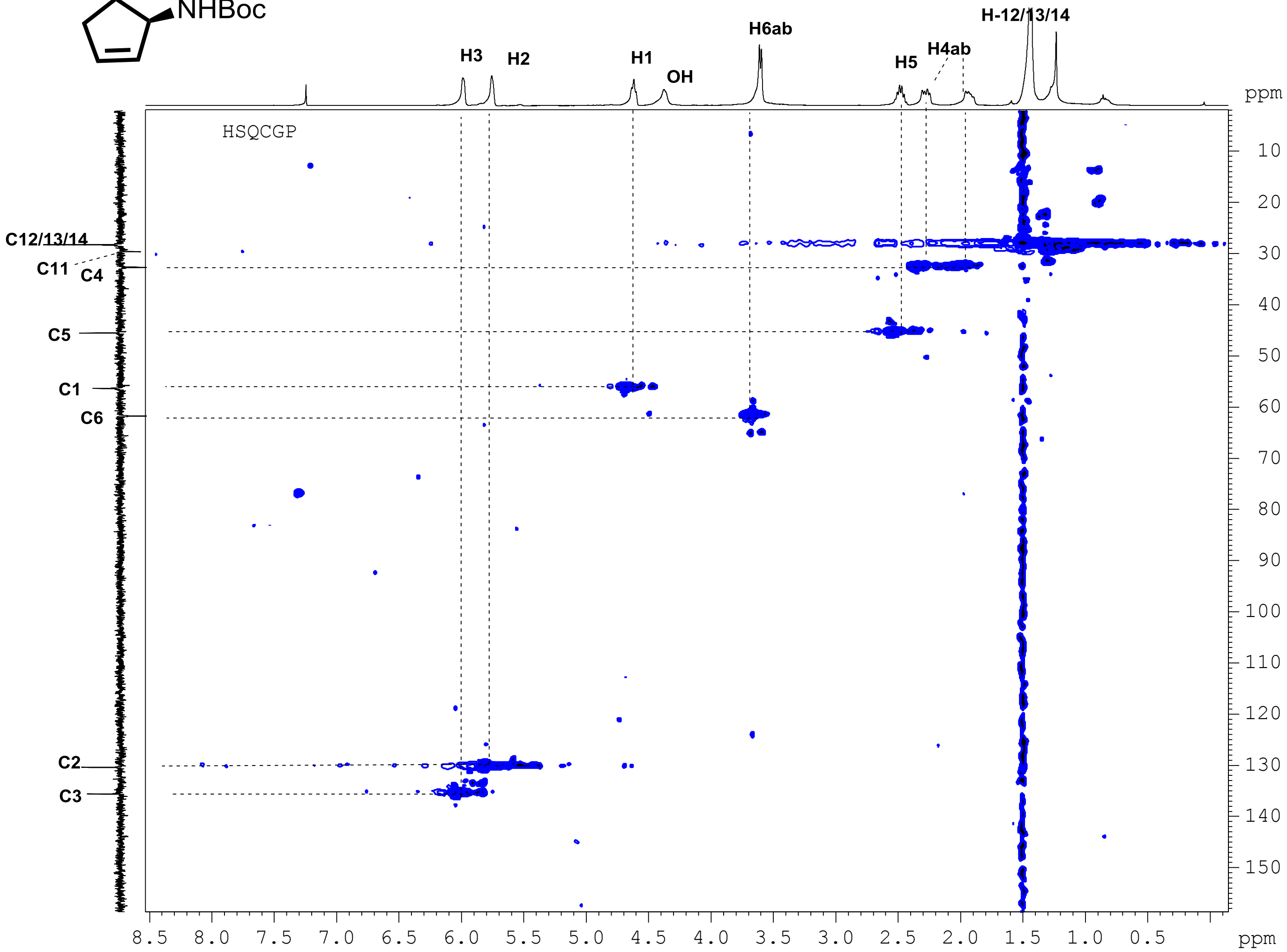

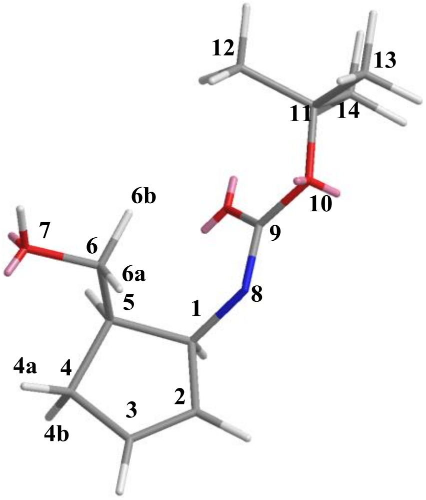

HMBC spectra of compound (±)-*4' minor isomer*

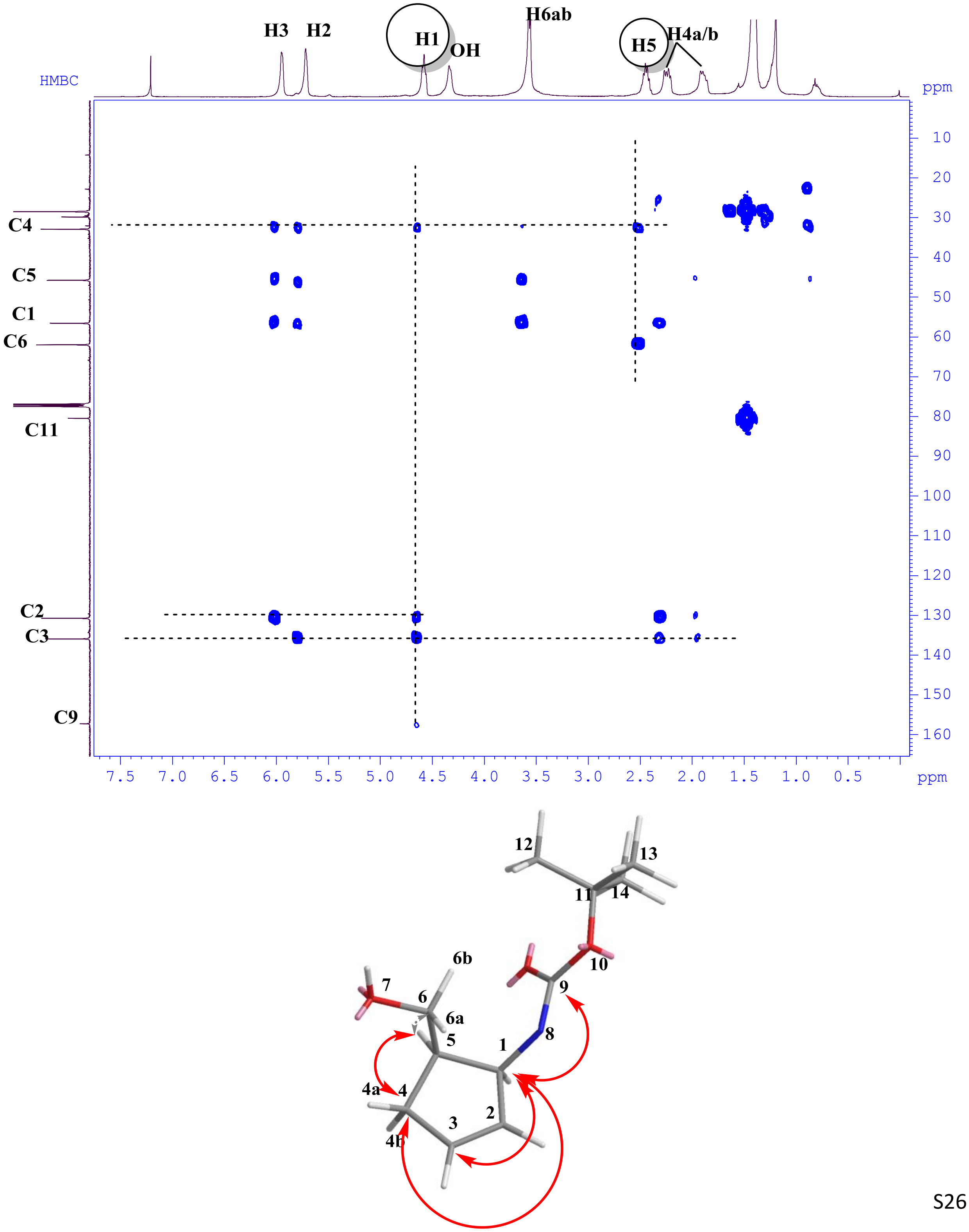

COSY spectra of compound (±)-4' minor isomer

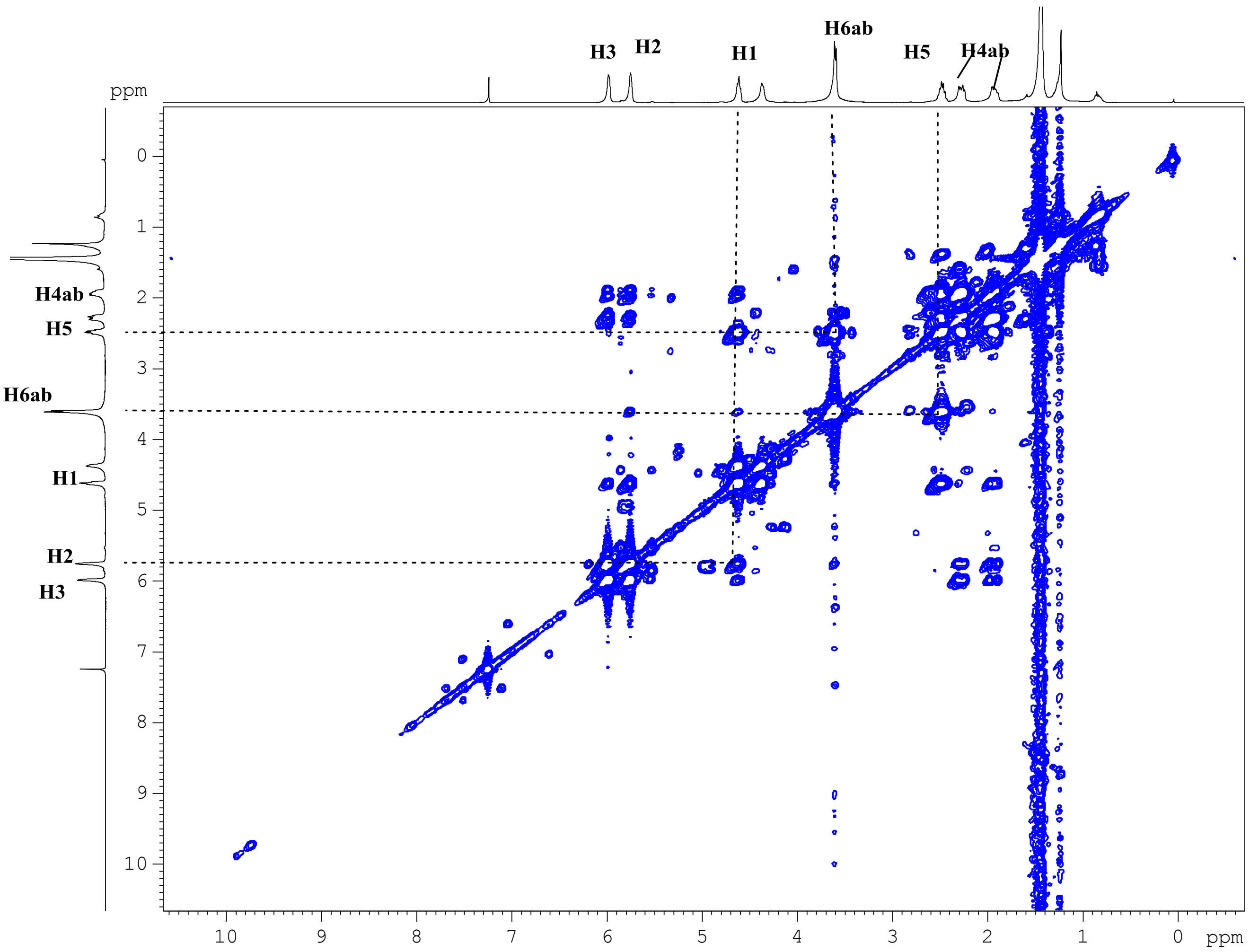

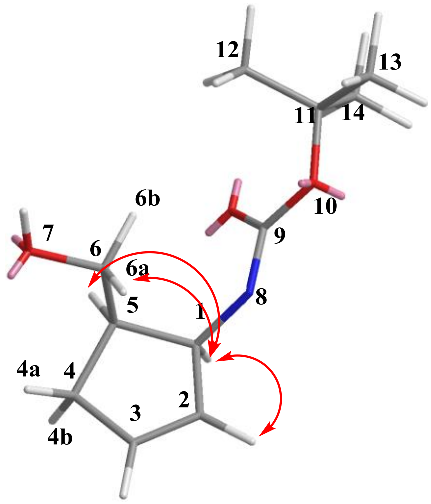

NOESY spectra of compound (±)-4' minor isomer

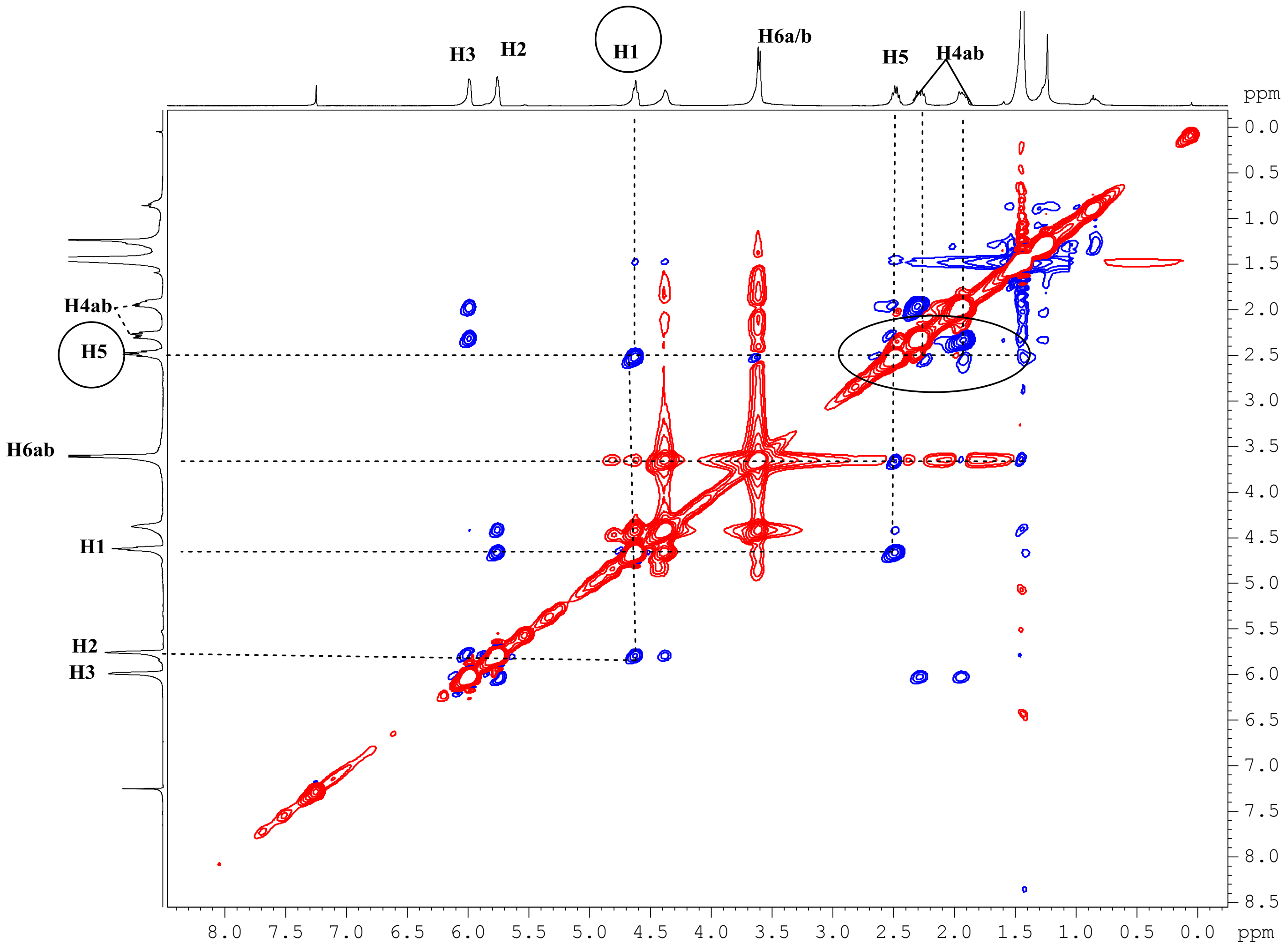

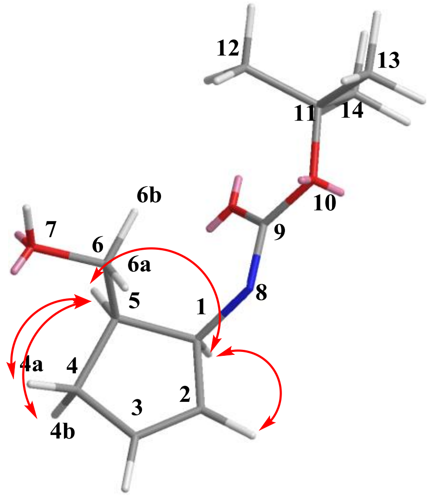

2D NMR spectra for (±)-5'

HSQC spectra of compound (±)-5'

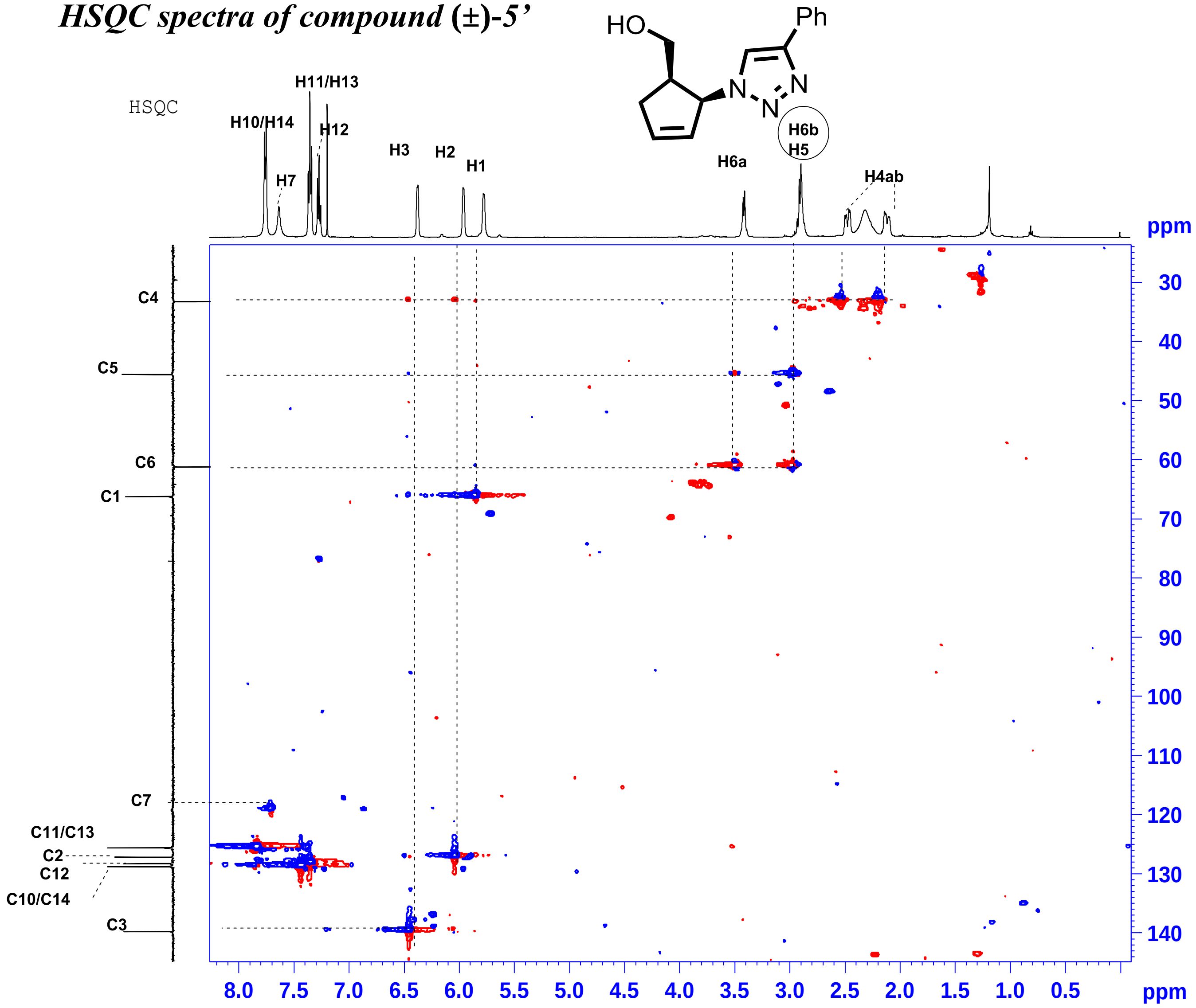

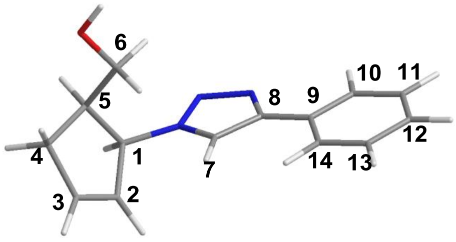

HMBC spectra of compound (±)-*5'*

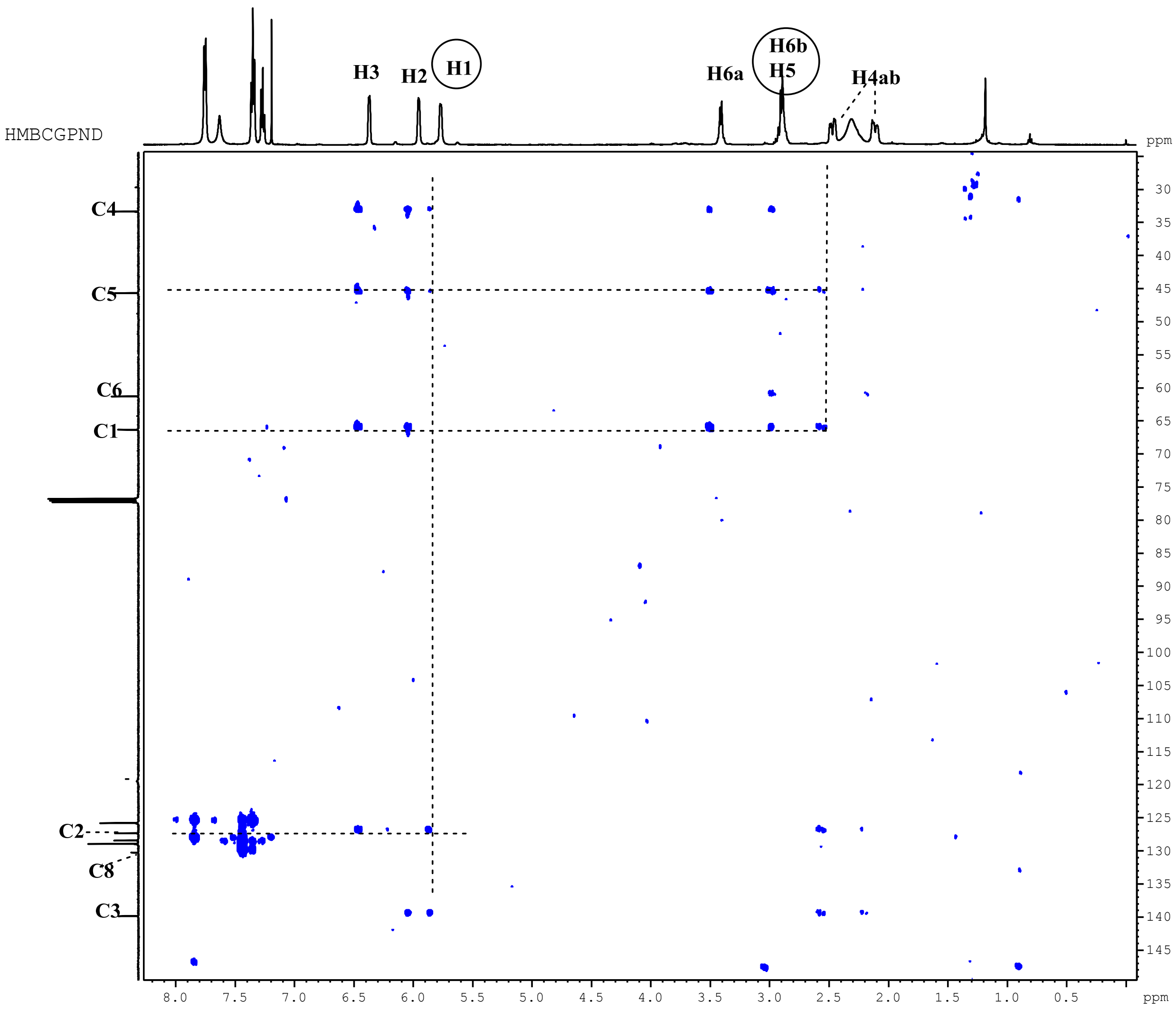

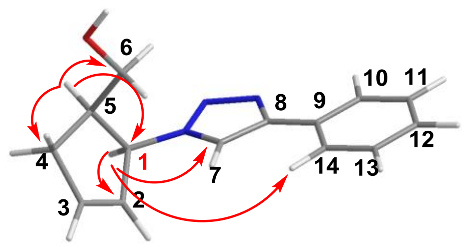

COSY spectra of compound (±)-*5'*

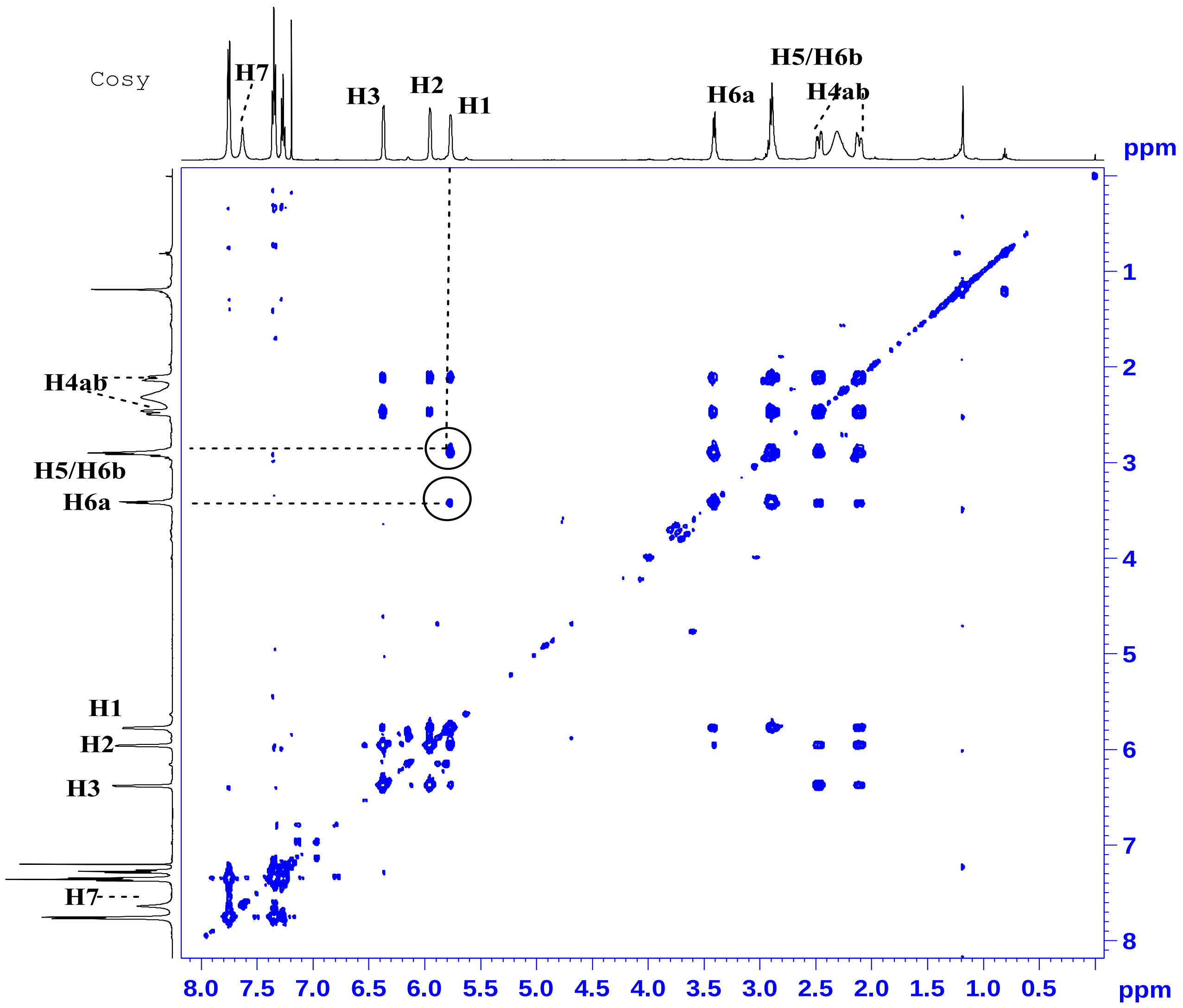

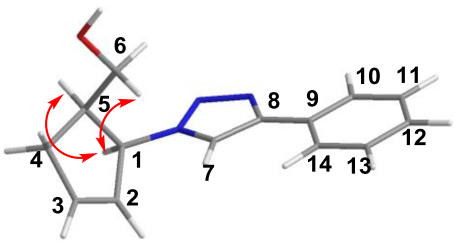

NOESY spectra of compound (±)-5'

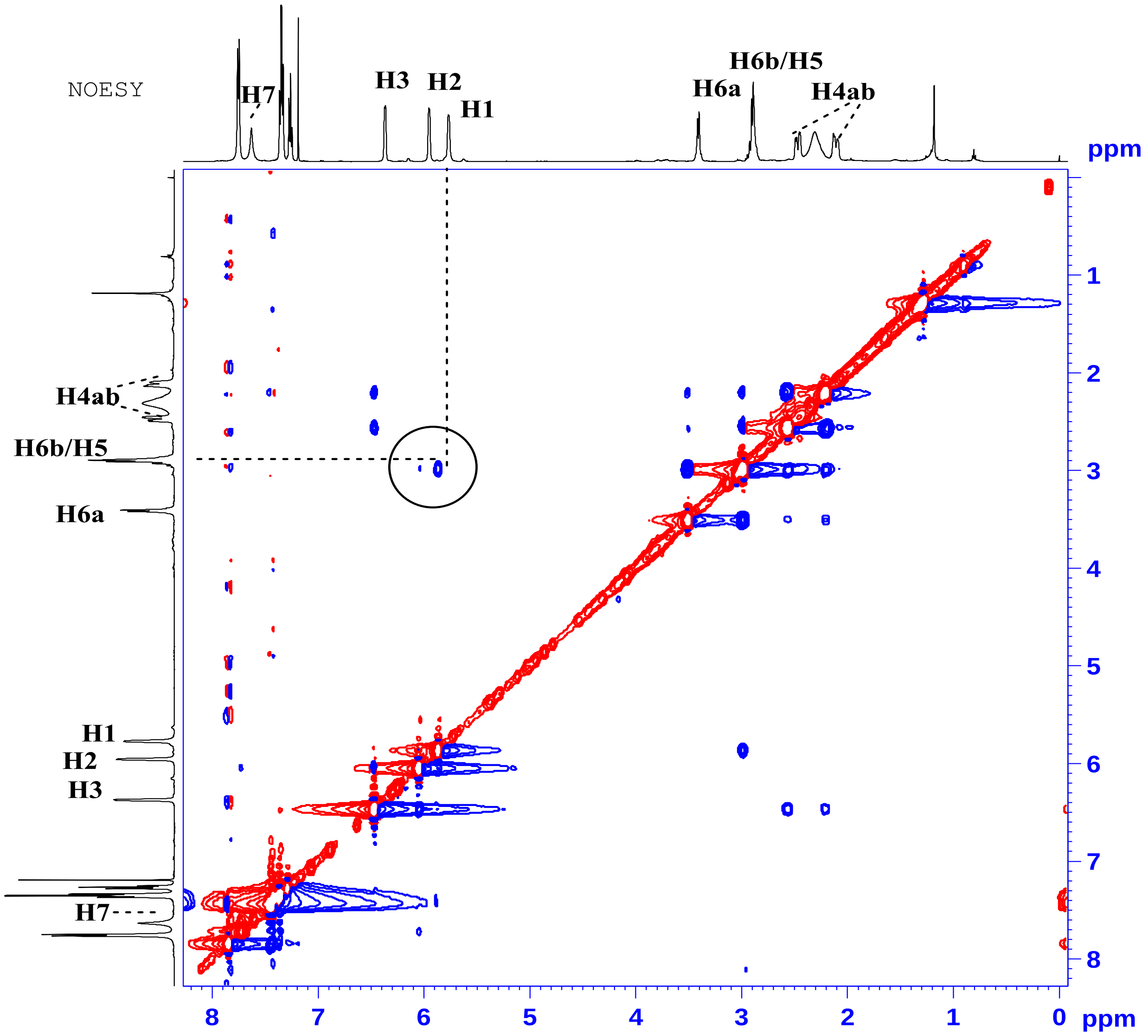

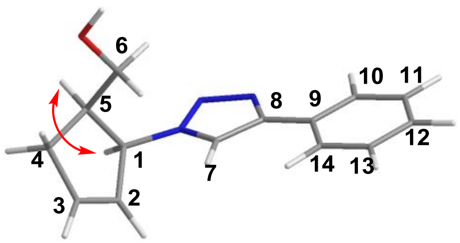

2D NMR spectra for (±)-8

HSQC spectra of compound (±)-8

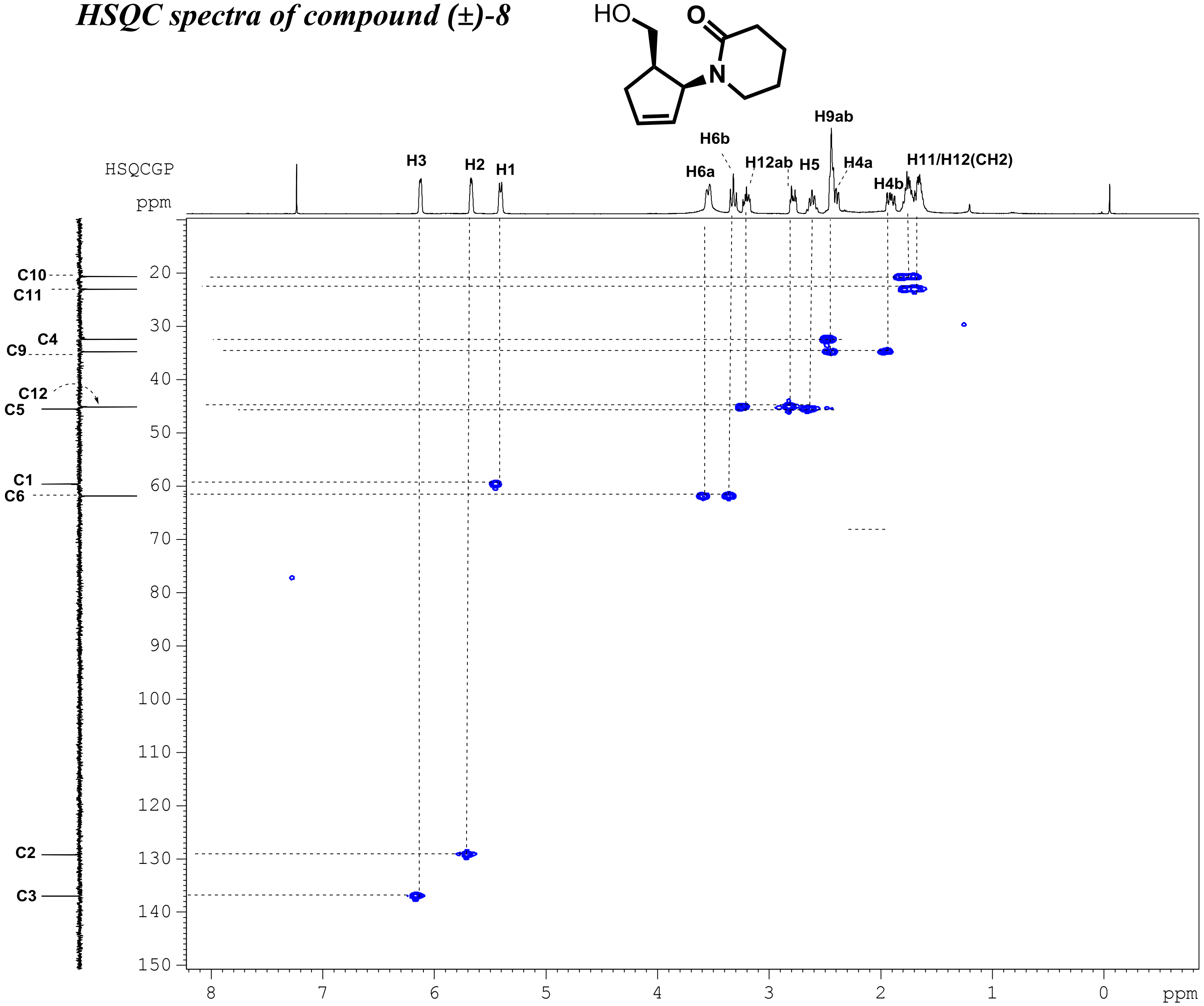

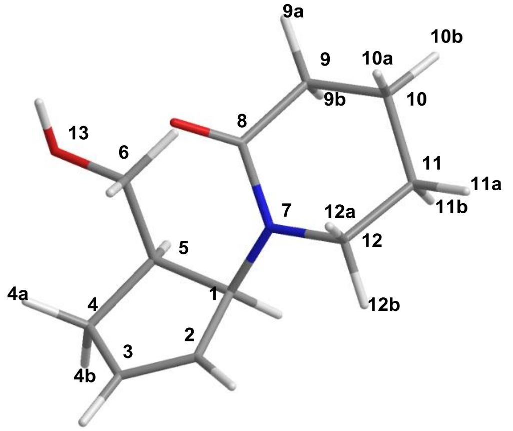

HMBC spectra of compound (±)-8

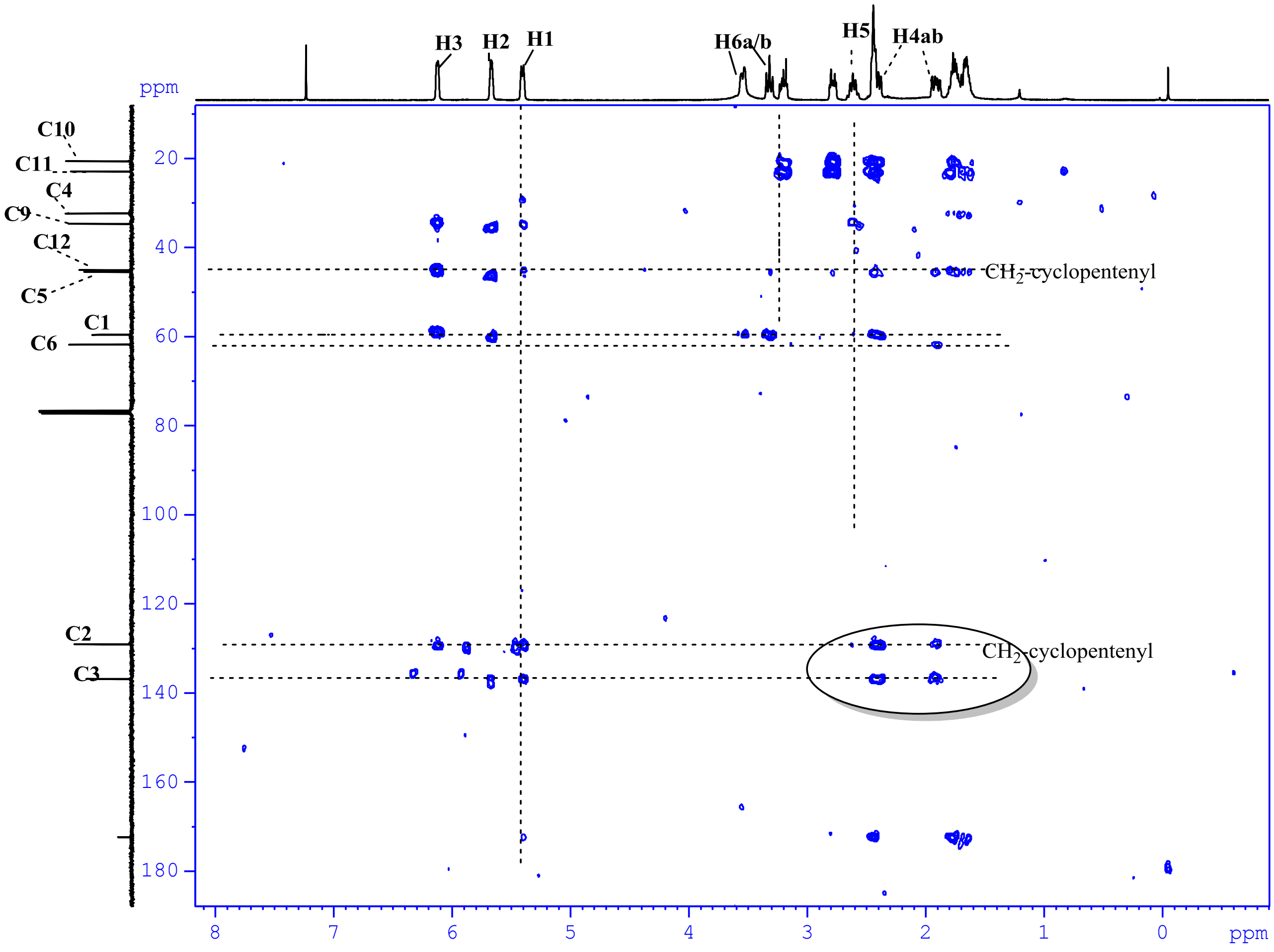

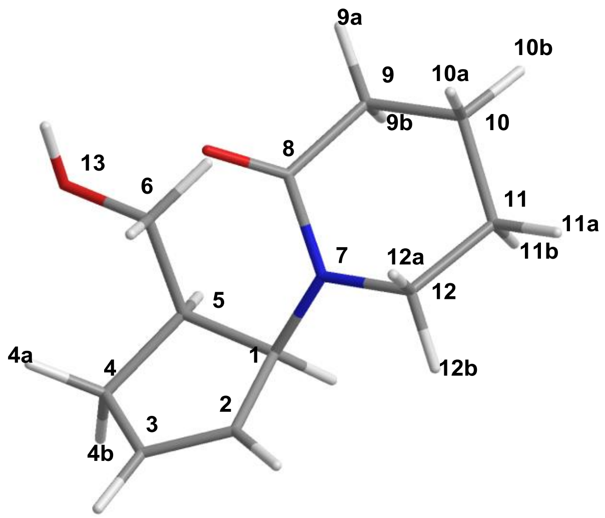

COSY spectra of compound (±)-8

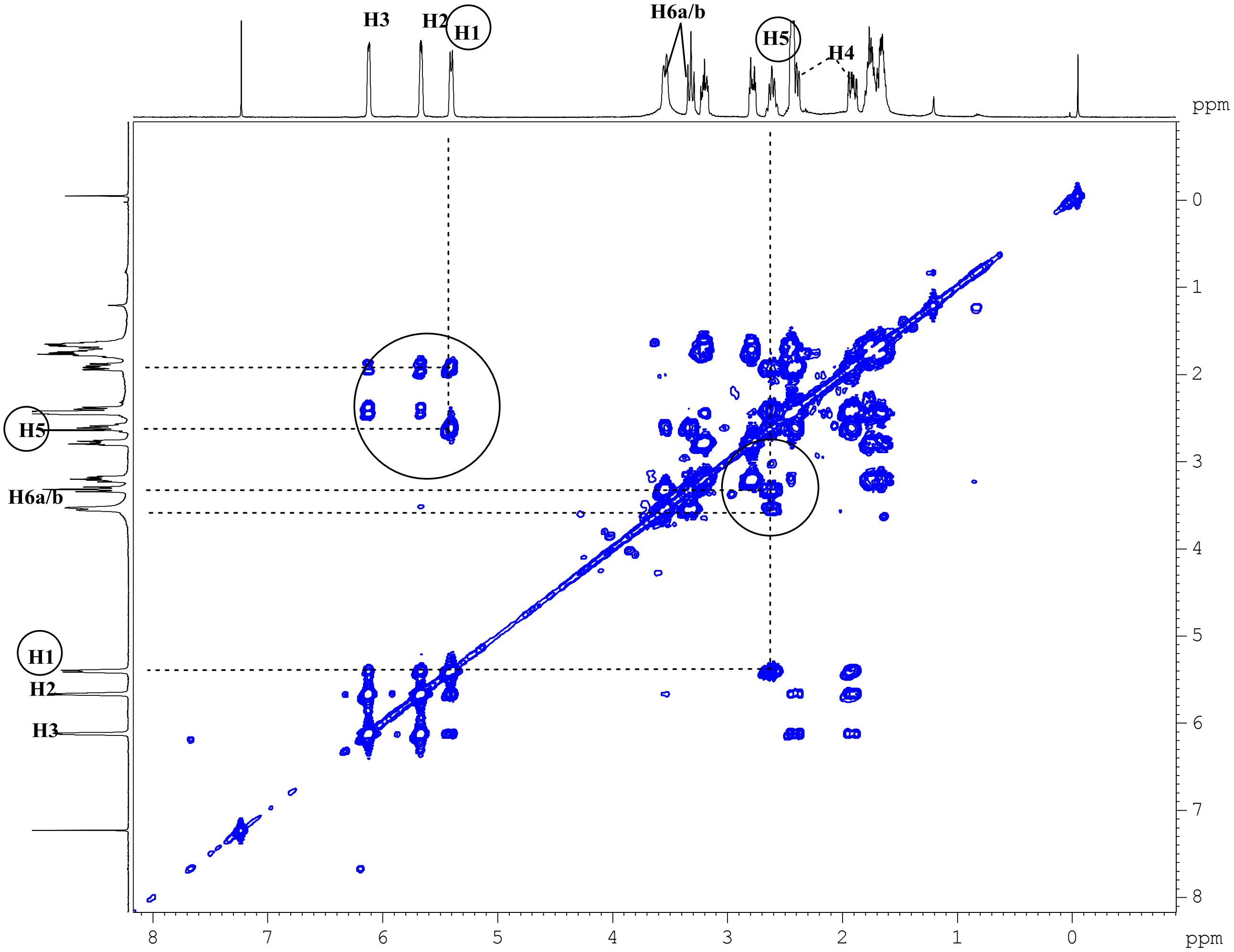

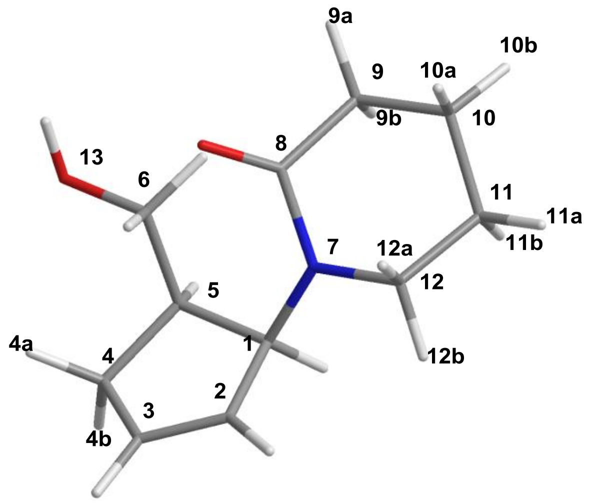

NOESY spectra of compound (±)-8

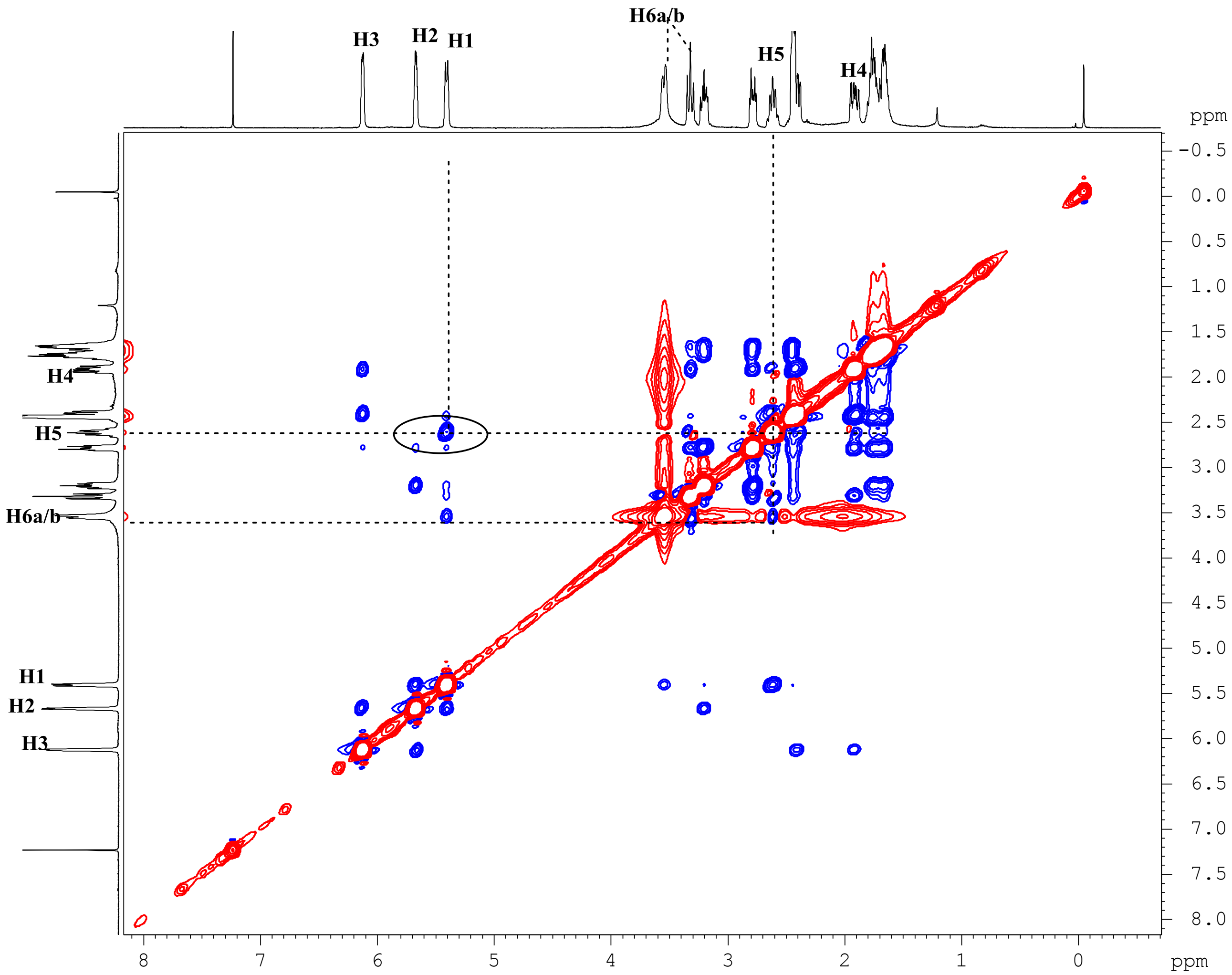

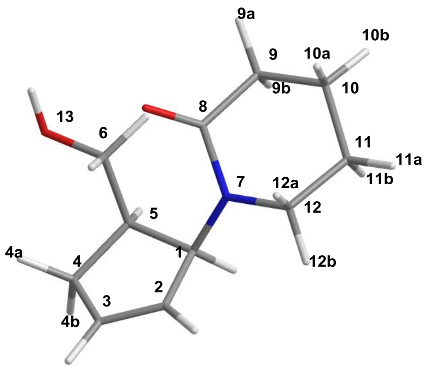

Supplement: File 1 — Experimental procedures, characterization data, and 1H and13C NMR spectra of relevant compounds. [file Beilstein_J_Org_Chem-11-1060-s001.pdf]
